# Supplementary material for: Bioactive Secondary Metabolites from an Arctic Marine-Derived Strain, Streptomyces sp. MNP-1, Using the OSMAC Strategy
Source: Molecules. 2025 Apr 8;30(8):1657. doi: 10.3390/molecules30081657 (PMC12029766; doi:10.3390/molecules30081657)
Supplement: Supplementary file 1 [file molecules-30-01657-s001.zip › molecules-3532373-supplementary.pdf]

Supplementary Material

Bioactive Secondary Metabolites from an Arctic Marine-derived Strain *Streptomyces* sp. MNP-1 using OSMAC Strategy

Mengna Wu [1], Zijun Liu [1], Jiahui Wang [1], Wentao Hu [2] and Huawei Zhang [1,*]

1 School of Pharmaceutical Sciences, Zhejiang University of Technology, Hangzhou 310014, China
2 College of Pharmaceutical Science & Collaborative Innovation Center of Yangtze River Delta Region Green Pharmaceuticals, Zhejiang University of Technology, Hangzhou 310014, China
* Correspondence: hwzhang@zjut.edu.cn (H.Z.); Fax: +86-571-88320913

CONTENTS

1. Experimental ... 4
1.1 Classification of Strain MNP-1 ... 4
1.2. Sequence Alignment ... 4
1.3. Phylogenetic Tree Construction ... 4
1.4.Small test medium preparation and fermentation ... 4
1.5.Prediction of the structure of the culture products of the small test ... 5
Table S1. Sequences producing significant alignments of strain MNP-1 ... 6
Table S2. Media for fermentation of strain MNP-1 ... 7
Table S3. Quality of crude extract of rice solid medium ... 8
Table S4. Quality of crude extract of Gauze's synthetic medium No.1(200 mL) ... 8
Table S5. Quality of crude extract of nutrient-rich liquid medium(200mL) ... 8
Table S6. Antimicrobial activity results of rice solid medium test ... 8
Table S7. Antimicrobial activity results of Gauze's synthetic medium No.1 test ... 9
Table S8. Antimicrobial activity of Gauze's synthetic medium No.1 fermentation time test ... 10
Table S9. Antimicrobial activity results of nutrient-rich liquid medium test ... 11
Figure S1. ^{1}H-NMR of rice solid medium test ... 12
Figure S2. HPLC peak shape of rice solid medium test ... 12
Figure S3. ^{1}H-NMR of Gauze's synthetic medium No.1 test ... 13
Figure S4. HPLC peak shape of Gauze's synthetic medium No.1 test ... 13
Figure S5. Fermentation morphology of Gauze's synthetic medium No.1 ... 14
Figure S6. ^{1}H-NMR of Gauze's synthetic medium No.1 fermentation time test ... 14
Figure S7. Antimicrobial activity of Gauze's synthetic medium No.1 fermentation time ... 14
Figure S8. ^{1}H-NMR of nutrient-rich liquid medium test ... 15
Figure S9. HPLC peak shape of nutrient-rich liquid medium test ... 15
Figure S10. Molecular network of nutrient-rich liquid medium test ... 16
Figure S11. ESI-MS spectrum of compound **1** ... 17
Figure S12. ^{1}H-NMR spectrum of compound **1** (CD_3OD, 600 MHz) ... 17
Figure S13. ^{13}C-NMR spectrum of compound **1**(CD_3OD, 150 MHz) ... 18
Figure S14. ESI-MS spectrum of compound **2** ... 19
Figure S15. ^{1}H-NMR spectrum of compound **2** ($CDCl_3$, 600 MHz) ... 19
Figure S16. ^{13}C-NMR spectrum of compound **2** ($CDCl_3$, 150 MHz) ... 20

Figure S17. HRESI-MS spectrum of compound **3** 21
Figure S18. ^{1}H-NMR spectrum of compound **3** ($CDCl_3$, 600 MHz) 21
Figure S19. HRESI-MS spectrum of compound **4** 22
Figure S20. ^{1}H-NMR spectrum of compound **4** (CD_3OD, 600 MHz) 22
Figure S21. ^{13}C-NMR spectrum of compound **4** (CD_3OD, 150 MHz) 23
Figure S22. HRESI-MS spectrum of compound **5** 24
Figure S23. ^{1}H-NMR spectrum of compound **5** (CD_3OD, 600 MHz) 24
Figure S24. HRESI-MS spectrum of compound **6** 25
Figure S25. ^{1}H-NMR spectrum of compound **6** (CD_3OD, 600 MHz) 25
Figure S26. ^{13}C-NMR spectrum of compound **6** (CD_3OD, 150 MHz) 26
Figure S27. HRESI-MS spectrum of compound **7** 27
Figure S28. ^{1}H-NMR spectrum of compound **7** (CD_3OD, 600 MHz) 27
Figure S29. HRESI-MS spectrum of compound **8** 28
Figure S30. ^{1}H-NMR spectrum of compound **8** (CD_3OD, 600 MHz) 28
Figure S31. ^{13}C-NMR spectrum of compound **8** (CD_3OD, 150 MHz) 29
Figure S32. HRESI-MS spectrum of compound **9** 30
Figure S33. ^{1}H-NMR spectrum of compound **9** (CD_3OD, 600 MHz) 30
Figure S34. ^{13}C-NMR spectrum of compound **9** (CD_3OD, 150 MHz) 31
Figure S35. HRESI-MS spectrum of compound **10** 32
Figure S36. ^{1}H-NMR spectrum of compound **10** (CD_3OD, 600 MHz) 32
Figure S37. ^{13}C-NMR spectrum of compound **10** (CD_3OD, 150 MHz) 33
Figure S38. HRESI-MS spectrum of compound **11** 34
Figure S39. ^{1}H-NMR spectrum of compound **11** (CD_3OD, 600 MHz) 34
Figure S40. ESI-MS spectrum of compound **12** 35
Figure S41. ^{1}H-NMR spectrum of compound **12** (CD_3OD, 600 MHz) 35
Figure S42. ^{13}C-NMR spectrum of compound **12** (CD_3OD, 150 MHz) 36
Figure S43. HRESI-MS spectrum of compound **13** 37
Figure S44. ^{1}H-NMR spectrum of compound **13** (CD_3OD, 600 MHz) 37
Figure S45. ^{13}C-NMR spectrum of compound **13** (CD_3OD, 150 MHz) 38
Figure S46. ESI-MS spectrum of compound **14** 39
Figure S47. ^{1}H-NMR spectrum of compound **14** (DMSO-d6, 600 MHz) 39
Figure S48. ^{13}C-NMR spectrum of compound **14** (DMSO-d6, 150 MHz) 40
Figure S49. HRESI-MS spectrum of compound **15** 41
Figure S50. ^{1}H-NMR spectrum of compound **15** ($CDCl_3$, 600 MHz) 41
Figure S51. ^{13}C-NMR spectrum of compound **15** ($CDCl_3$, 150 MHz) 42
Figure S52. HRESI-MS spectrum of compound **16** 43
Figure S53. ^{1}H-NMR spectrum of compound **16** ($CDCl_3$, 600 MHz) 43
Figure S54. ^{13}C-NMR spectrum of compound **16** ($CDCl_3$, 150 MHz) 44
Figure S55. HRESI-MS spectrum of compound **17** 45
Figure S56. ^{1}H-NMR spectrum of compound **17** ($CDCl_3$, 600 MHz) 45
Figure S57. ^{13}C-NMR spectrum of compound **17** ($CDCl_3$, 150 MHz) 46
Figure S58. ESI-MS spectrum of compound **18** 47
Figure S59. ^{1}H-NMR spectrum of compound **18** (CD_3OD, 600 MHz) 47
Figure S60. ^{13}C-NMR spectrum of compound **18** (CD_3OD, 150 MHz) 48

Figure S61. ESI-MS spectrum of compound **19** 49
Figure S62. ^{1}H-NMR spectrum of compound **19** (CD_3OD, 600 MHz) 49
Figure S63. ^{13}C-NMR spectrum of compound **19** (CD_3OD, 150 MHz) 50
Figure S64. ESI-MS spectrum of compound **20** 51
Figure S65. ^{1}H-NMR spectrum of compound **20** (CD_3OD, 600 MHz) 51
Figure S66. ^{13}C-NMR spectrum of compound **20** (CD_3OD, 150 MHz) 52
Figure S67. morphology of the ore sample from Arctic origin 52
References 53

1. Experimental

1.1 Classification of Strains

Fresh organisms of strain MNP-1 were entrusted to Beijing Kengke Biotechnology Co., Ltd, Hangzhou Branch, for 16S rRNA sequence sequencing. The sequencing results were spliced using the software Contig Express. Inaccurate parts at both ends were removed, and the total length of the spliced 16*S* rRNA sequences was 1423 bp, which were as follows:

GGTGGCGGGTGCTACCATGCAGTCGAACGATGAAGCCGCTTCGGTGGTGGATTAG-
TGGCGAACGGGTGAGTAACACGTGGGCAATCTGCCCTTCACTCTGGGACAAGCCCTGGAAACGGGGTCTAAT
ACCGGATAACACTCTGTCCCGCATGGGACGGGGTTAAAA-
GCTCCGGCGGTGAAGGATGAGCCCGCGGCCTATCAGCTTGTTGGTGGGGTAATGGCCTACCAAGGCGACGAC
GGGTAGCCGGCCTGAGAGGGCGACCGGCCACACTGGGACTGAGACACGGCCCAGACTCCTAC-
GGGAGGCAGCAGTGGGGAATATTGCACAATGGGCGAAAGCCTGATGCAGCGACGCCGCGTGAGGGATGAC
GGCCTTCGGGTTGTAAACCTCTTTCAGCAGGGAAGAAGCGAAAGTGACGGTACCTGCAGAA-
GAAGCGCCGGCTAACTACGTGCCAGCAGCCGCGGTAATACGTAGGGCGCAAGCGTTGTCCGGAATTATTGGG
CGTAAAGAGCTCGTAGGCGGCTTGTCACGTCGGATGTGAAA-
GCCCGGGGCTTAACCCCGGGTCTGCATTCGATACGGGCTAGCTAGAGTGTGGTAGGGGAGATCGGAATTCCT
GGTGTAGCGGTGAAATGCGCAGATATCAGGAGGAACACCGGTGGCGAAGGCG-
GATCTCTGGGCCATTACTGACGCTGAGGAGCGAAAGCGTGGGGAGCGAACAGGATTAGATACCCTGGTAGTC
CACGCCGTAAACGTTGGGAACTAGGTGTTGGCGACATTCCACGTCGTCGGTGCCG-
CAGCTAACGCATTAAGTTCCCCGCCTGGGGAGTACGGCCGCAAGGCTAAAACTCAAAGGAATTGACGGGGG
CCCGCACAAGCAGCGGAGCATGTGGCTTAATTCGACGCAACGCGAAGAACCTTAC-
CAAGGCTTGACATATACCGGAAAGCATCAGAGATGGTGCCCCCCTTGTGGTCGGTATACAGGTGGTGCATGG
CTGTCGTCAGCTCGTGTCGTGAGATGTTGGGTTAAGTCCCGCAACGAGCGCAACCCTT-
GTTCTGTGTTGCCAGCATGCCCTTCGGGGTGATGGGGACTCACAGGAGACTGCCGGGGTCAACTCGGAGGAA
GGTGGGGACGACGTCAAGTCATCATGCCCCTTATGTCTTGGGCTGCACACGTGC-
TACAATGGCCGGTACAATGAGCTGCGATGCCGCGAGGCGGAGCGAATCTCAAAAAGCCGGTCTCAGTTCGG
ATTGGGGTCTGCAACTCGACCCCATGAAGTCGGAGTTGCTAGTAATCGCAGATCAGCATT-
GCTGCGGTGAATACGTTCCCGGGCCTTGTACACACCGCCCGTCACGTCACGAAAGTCGGTAACACCCGAAGC
CGGTGGCCCAACCCCTTGTGGGAGGGAGCTTCGAAGGTGACGA.

1.2. Sequence Alignment

The 16S rRNA sequence of strain MNP-1 was uploaded to the NCBI database for nucleic acid sequence comparison, and the specific information of similarity ten. (TableS1) The results showed that strain MNP-1 belonged to the genus *Streptomyces* and had the highest degree of homology of 99.85% with the strain *Streptomyces pratensis* ch24 in the NCBI database (accession number: NR 125616.1) in the NCBI database, which has the highest degree of similarity (99.85%), but a lower Total Score.The next highest rank of similarity was strain *Streptomyces acrimycini* CSSP430 (accession number: NR_115449.1) with 99.79% similarity.

1.3. Phylogenetic Tree Construction

According to the results of nucleic acid sequence comparison, the 16S rRNA sequences of 30 strains with high degree of homology were downloaded, and the strain *Streptosporangium becharense* DSM46887, which is in the same family of *Streptomyces* but in a different genus as strain MNP-1, was selected as the exogenous strain, and the strains were clustered using the MEGA software (7.0.26), and a phylogenetic tree was constructed.(**Figure 3**) The topology of the phylogenetic tree showed that strain MNP-1 and *S. acrimycini* CSSP430 (accession number: NR_115449.1) were clustered together with a Bootstrap value of 94%. Combined with the BLAST comparison results, it was initially considered that *Streptomyces* MNP-1 was the closest interspecific relative to *S. acrimycini.*

1.4.Small test medium preparation and fermentation

In order to determine the optimal levels of inducers in the medium, the concentration gradients of $CuSO_4$-5H_2O were set as no additive, 20 mg/L, 40 mg/L and 60 mg/L (#1-#4), and the concentration gradients of epigenetic modifiers were set as no additive, 10 μM, 50 μM, 100 μM and 200 μM (#5-#13). In addition five other nutrient-enriched liquid media were selected for small trials (#14-#18). All media were fermented in parallel in three flasks.(**Table.S2**)

1.5.Prediction of the structure of the culture products of the small test

The concentration of methanol solution of crude extract was 1 mg/mL, and the samples were detected by UHPLC-Q-TOF-MS/MS using SCIEX X500B QTOF high-resolution triple quadrupole tandem time-of-flight mass spectrometry coupled with SCIEX ExionLC ultra-high performance liquid chromatograph. The chromatographic column (Phenomenex Kinetex, C18, 150×2.1 mm, 5 μm,CH_3OH-H_2O, 10:90-100:0, v/v, 0.3 mL/min), gradient elution; the mass spectrometry conditions were electrospray ionization (ESI), positive and negative ions in full scanning mode, with a scanning range m/ z 100-2000 (MS), 50-2000 (MS/MS). The data were converted to .mzXML format using MSconvert software, and the files were uploaded to the Global Natural Products Molecular Network (GNPS, https://gnps.ucsd.edu) for mass spectral fragment ion similarity comparison and molecular network mapping via the FTP client software FileZilla. Data visualization and node annotation using Cytoscape software system

Table S1. Sequences producing significant alignments of strain MNP-1

Accession	Description	Max Score	Total Score	Query Coverage	Per. Ident	Acc. Len
NR_125616.1	*Streptomyces pratensis* strain ch24 16S ribosomal RNA	2521	2521	96%	99.85%	1370
NR_115449.1	*Streptomyces acrimycini* strain CSSP430 16S ribosomal RNA	2573	2573	98%	99.79%	1407
NR_118107.1	*Streptomyces globisporus* strain KCTC 9026 16S ribosomal RNA	2562	2562	98%	99.64%	1403
NR_115450.1	*Streptomyces griseinus* strain CSSP433 16S ribosomal RNA	2547	2547	97%	99.64%	1402
NR_114493.1	*Streptomyces caviscabies* strain ATCC 51928 16S ribosomal RNA	2590	2590	99%	99.58%	1523
NR_043351.1	*Streptomyces fimicarius* strain CSSP537 16S ribosomal RNA	2590	2590	99%	99.58%	1496
NR_112591.1	*Streptomyces flavofuscus* strain NBRC 100768 16S ribosomal RNA	2590	2590	99%	99.58%	1483
NR_112440.1	*Streptomyces baarnensis* strain NBRC 14727 16S ribosomal RNA	2590	2590	99%	99.58%	1479
NR_112347.1	*Streptomyces fimicarius* strain NBRC 13037 16S ribosomal RNA	2590	2590	99%	99.58%	1474
NR_112334.1	*Streptomyces sindenensis* strain CSSP727 16S ribosomal RNA	2580	2580	99%	99.44%	1422

Table S2. Media for fermentation of strain MNP-1

No.	Media component	Liquid /Solid	Time(Days)
#1	rice 80 g/L, rice:H_2O=2:3 (g/mL)	S	30
#2	rice 80 g/L, $CuSO_4{\cdot}5H_2O$ 20 mg/L, rice:H_2O=2:3 (g/mL)	S	30
#3	rice 80 g/L, $CuSO_4{\cdot}5H_2O$ 40 mg/L, rice:H_2O=2:3 (g/mL)	S	30
#4	rice 80 g/L, $CuSO_4{\cdot}5H_2O$ 60 mg/L, rice:H_2O=2:3 (g/mL)	S	30
#5	soluble starch 20.0 g, NaCl 0.5 g, $K_2HPO_4{\cdot}3H_2O$ 0.5 g, KNO_3 1.0 g, $FeSO_4{\cdot}7H_2O$ 0.01 g, $MgSO_4{\cdot}7H_2O$ 0.5 g, H_2O 1000 mL, pH 7.0	S	14
#6	#5 supplemented with 10 μM 5-Aza-C	L	14
#7	#5 supplemented with 50 μM 5-Aza-C	L	7
#8	#5 supplemented with 100 μM 5-Aza-C	L	14
#9	#5 supplemented with200 μM 5-Aza-C	L	14
#10	#5 supplemented with 10 μM SAHA	L	14
#11	#5 supplemented with 50 μM SAHA	L	14
#12	#5 supplemented with 100 μM SAHA	L	14
#13	#5 supplemented with 200 μM SAHA	L	14
#14 ISP-2	Yeast extract 4.0 g, malt extract 10.0 g, glucose 4.0 g, H_2O 1000 mL	L	14
#15 YMG	Glucose 10.0 g, soluble starch 20.0 g, sea salt 30.0 g, malt extract 5.0 g, $CaCO_3$ 0.5 g, yeast extract 5.0 g, H_2O 1000 mL	L	14
#16 MC1	Soluble starch 35.0 g, glucose 10.0 g, beef paste 3.5 g, acid hydrolyzed casein 2.0 g, yeast extract 20.0 g, soybean meal 10.0 g, $CaCO_3$ 2.0 g, H_2O 1000 mL	L	14
#17 YPM	mannitol 4.0 g, yeast extract 2.0 g, peptone 2.0 g, artificial seawater 1000 mL	L	14
#18 312	10.0 g soluble starch, 10.0 g glucose, 10.0 g glycerol, 5.0 g peptone, 2.0 g yeast extract, 2.5 g corn starch, 3.0 g $CaCO_3$, H_2O 1000 mL	L	14

Table S3. Quality of crude extract of rice solid medium

No.	#1	#2	#3	#4
m(g)	0.0541	0.0682	0.0511	0.0487

Table S4. Quality of crude extract of Gauze's synthetic medium No.1(200 mL)

No.	#5	#6	#7	#8	#9	#10	#11	#12	#13
m(g)	0.0147	0.0208	0.0202	0.0144	0.0204	0.0436	0.0140	0.0323	0.0185

Table S5. Quality of crude extract of nutrient-rich liquid medium(200mL)

No.	#14	#15	#16	#17	#18
m(g)	0.0432	0.1012	0.1570	0.0482	0.0861

Table S6. Antimicrobial activity results of rice solid medium test

No.	Concentration	Inhibitory circle diameter（mm）		
		S. aureus	*E. coli*	*C. albicans*
#1	100 mg/mL	12	-	-
	10 mg/mL	8	-	-
	1 mg/mL	-	-	-
#2	100 mg/mL	14	10	-
	10 mg/mL	9	-	-
	1 mg/mL	-	-	-
#3	100 mg/mL	9	7	-
	10 mg/mL	-	-	-
	1 mg/mL	-	-	-
#4	100 mg/mL	8	-	-
	10 mg/mL	-	-	-
	1 mg/mL	-	-	-
Positive Control	1mg/mL	26	12	11
Negative Control		-	-	-

Table S7. Antimicrobial activity results of Gauze's synthetic medium No.1 test

No.	Concentration	Inhibitory circle diameter（mm）		
		S. aureus	*E. coli*	*C. albicans*
#5	100 mg/mL	10	-	-
	10 mg/mL	-	-	-
	1 mg/mL	-	-	-
#6	100 mg/mL	10	-	-
	10 mg/mL	-	-	-
	1 mg/mL	-	-	-
#7	100 mg/mL	9	-	-
	10 mg/mL	-	-	-
	1 mg/mL	-	-	-
#8	100 mg/mL	7	-	-
	10 mg/mL	-	-	-
	1 mg/mL	-	-	-
#9	100 mg/mL	-	-	-
	10 mg/mL	-	-	-
	1 mg/mL	-	-	-
#10	100 mg/mL	-	-	-
	10 mg/mL	-	-	-
	1 mg/mL	-	-	-
#11	100 mg/mL	-	-	-
	10 mg/mL	-	-	-
	1 mg/mL	-	-	-
#12	100 mg/mL	-	-	-
	10 mg/mL	-	-	-
	1 mg/mL	-	-	-
#13	100 mg/mL	-	-	-
	10 mg/mL	-	-	-
	1 mg/mL	-	-	-
Positive Control	1 mg/mL	28	12	11
Negative Control		-	-	-

Table S8. Antimicrobial activity of Gauze's synthetic medium No.1 fermentation time test

No.	Concentration	Inhibitory circle diameter（mm）		
		S. aureus	*E. coli*	*C. albicans*
#7 medium 7 d	100 mg/mL	19	-	-
	10 mg/mL	16	-	-
	1 mg/mL	12	-	-
#7 medium 10 d	100 mg/mL	8	-	-
	10 mg/mL	-	-	-
	1 mg/mL	-	-	-
#7 medium 14 d	100 mg/mL	8	-	-
	10 mg/mL	-	-	-
	1 mg/mL	-	-	-
#7 medium 20 d	100 mg/mL	7	-	-
	10 mg/mL	-	-	-
	1 mg/mL	-	-	-
Positive Control	1 mg/mL	31	12	12
Negative Control		-	-	-

Table S9. Antimicrobial activity results of nutrient-rich liquid medium test

No.	Concentration	Inhibitory circle diameter（mm）		
		S. aureus	*E. coli*	*C. albicans*
#14	100 mg/mL	8	-	-
	10 mg/mL	-	-	-
	1 mg/mL	-	-	-
#15	100 mg/mL	8	9	-
	10 mg/mL	-	-	-
	1 mg/mL	-	-	-
#16	100 mg/mL	9	8	-
	10 mg/mL	7	-	-
	1 mg/mL	-	-	-
#17	100 mg/mL	7	-	-
	10 mg/mL	-	-	-
	1 mg/mL	-	-	-
#18	100 mg/mL	7	-	-
	10 mg/mL	-	-	-
	1 mg/mL	-	-	-
Positive Control	1 mg/mL	21	12	12
Negative Control		-	-	-

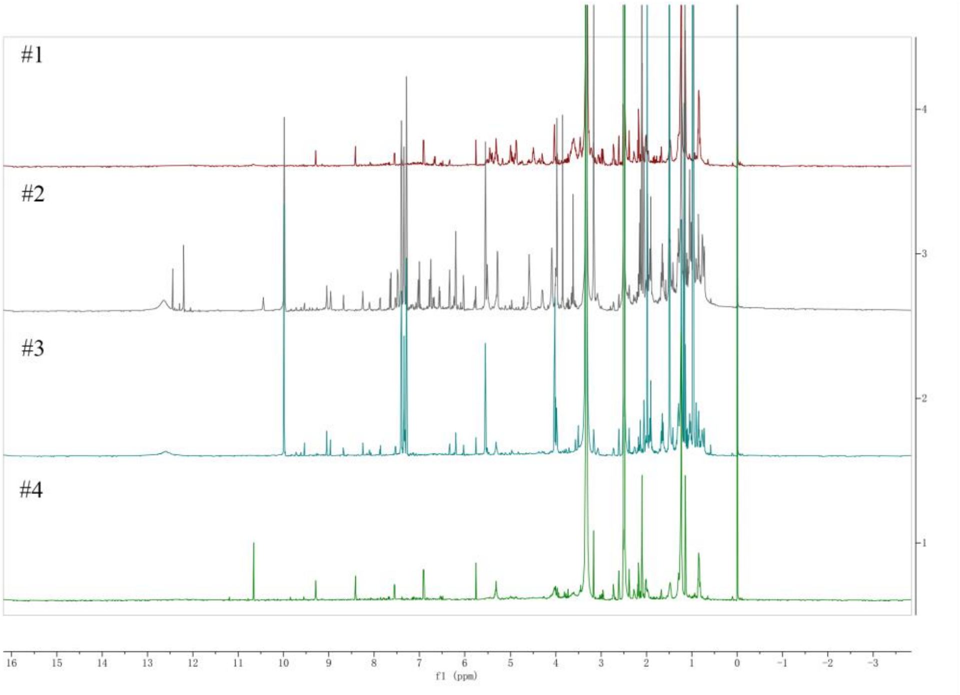

Figure S1. ^{1}H-NMR of rice solid medium test

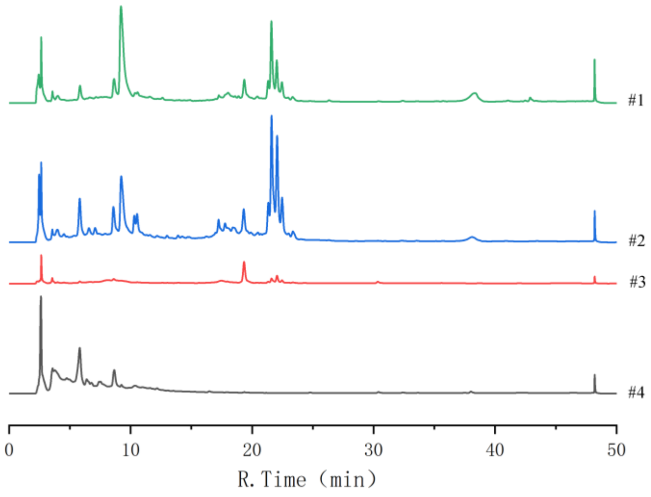

Figure S2. HPLC peak shape of rice solid medium test

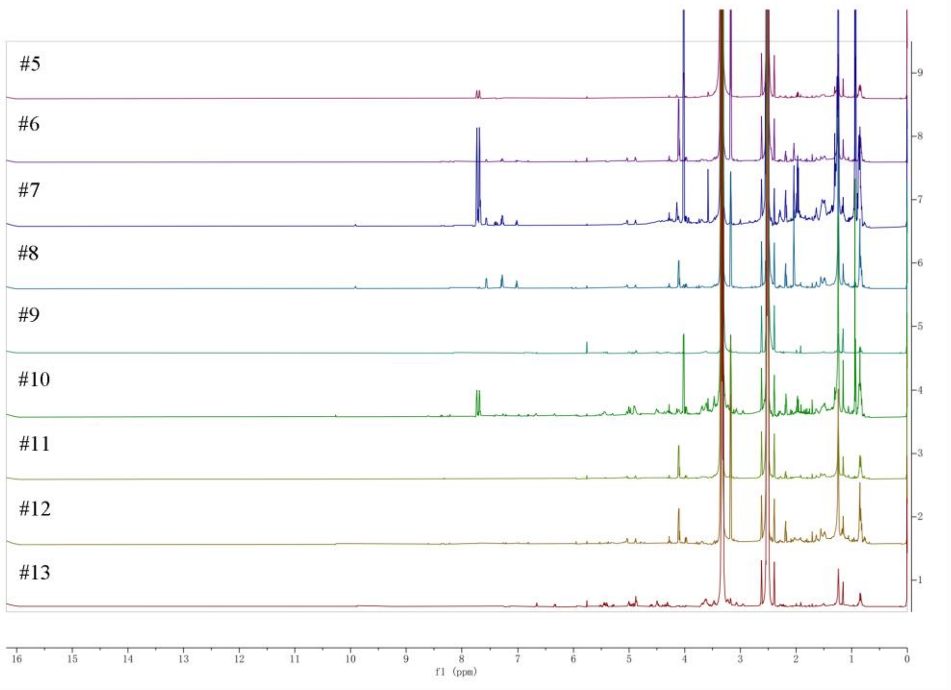

Figure S3. ^{1}H-NMR of Gauze's synthetic medium No.1 test

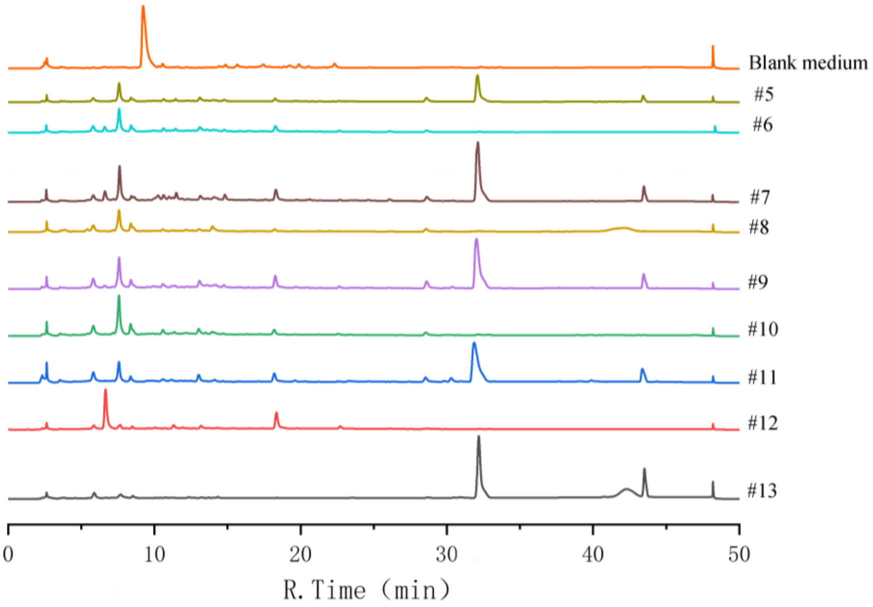

Figure S4. HPLC peak shape of Gauze's synthetic medium No.1 test

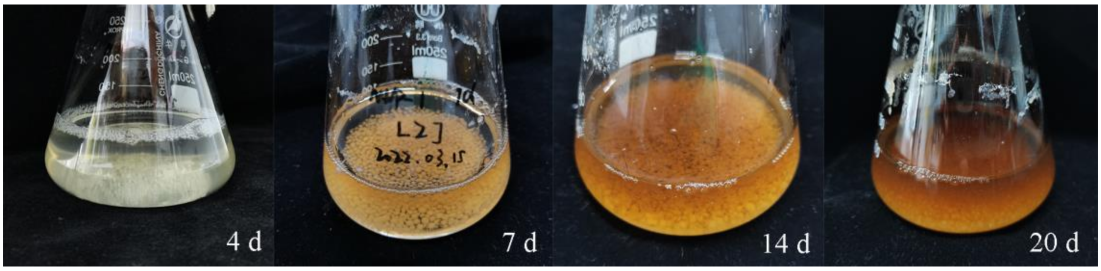

Figure S5. Fermentation morphology of Gauze's synthetic medium No.1

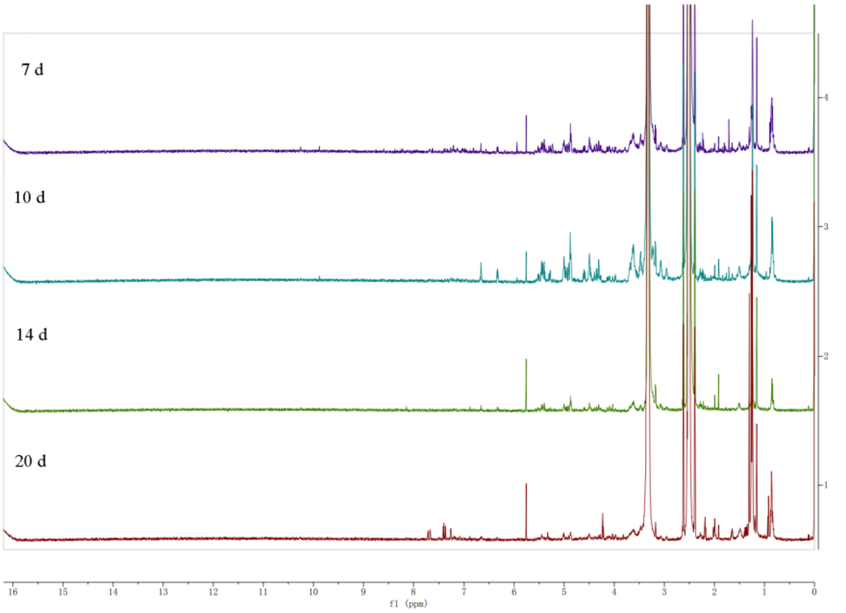

Figure S6. ^{1}H-NMR of Gauze's synthetic medium No.1 fermentation time test

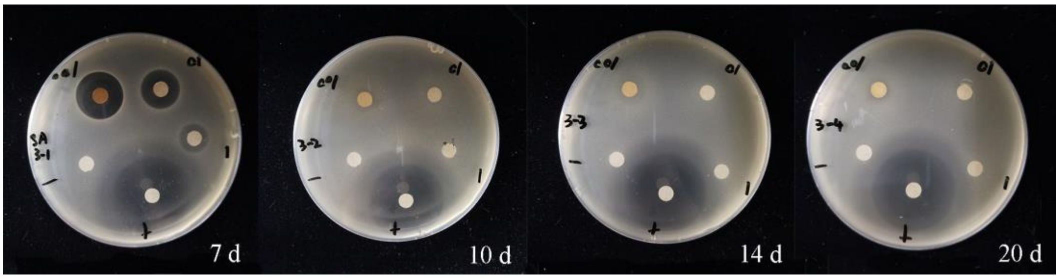

Figure S7. Antimicrobial activity of Gauze's synthetic medium No.1 fermentation time

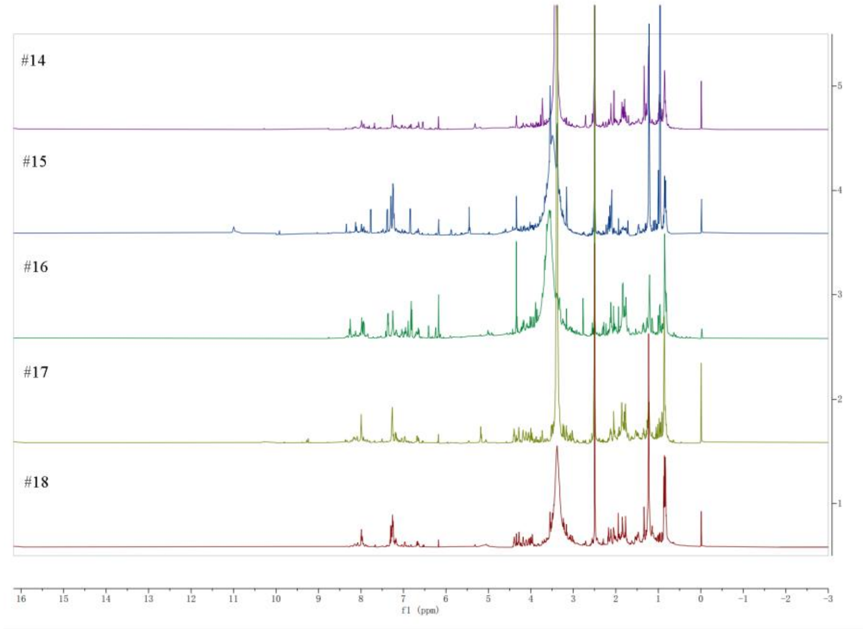

Figure S8. ^{1}H-NMR of nutrient-rich liquid medium test

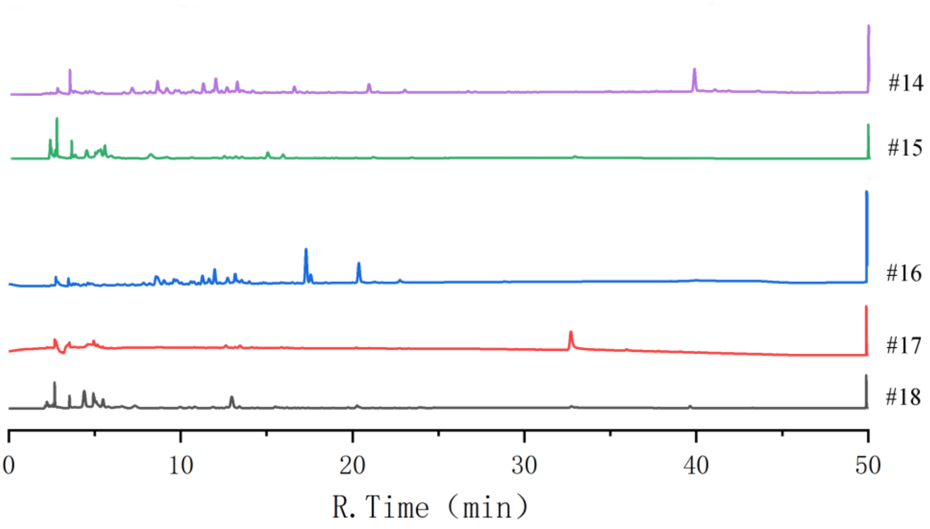

Figure S9. HPLC peak shape of nutrient-rich liquid medium test

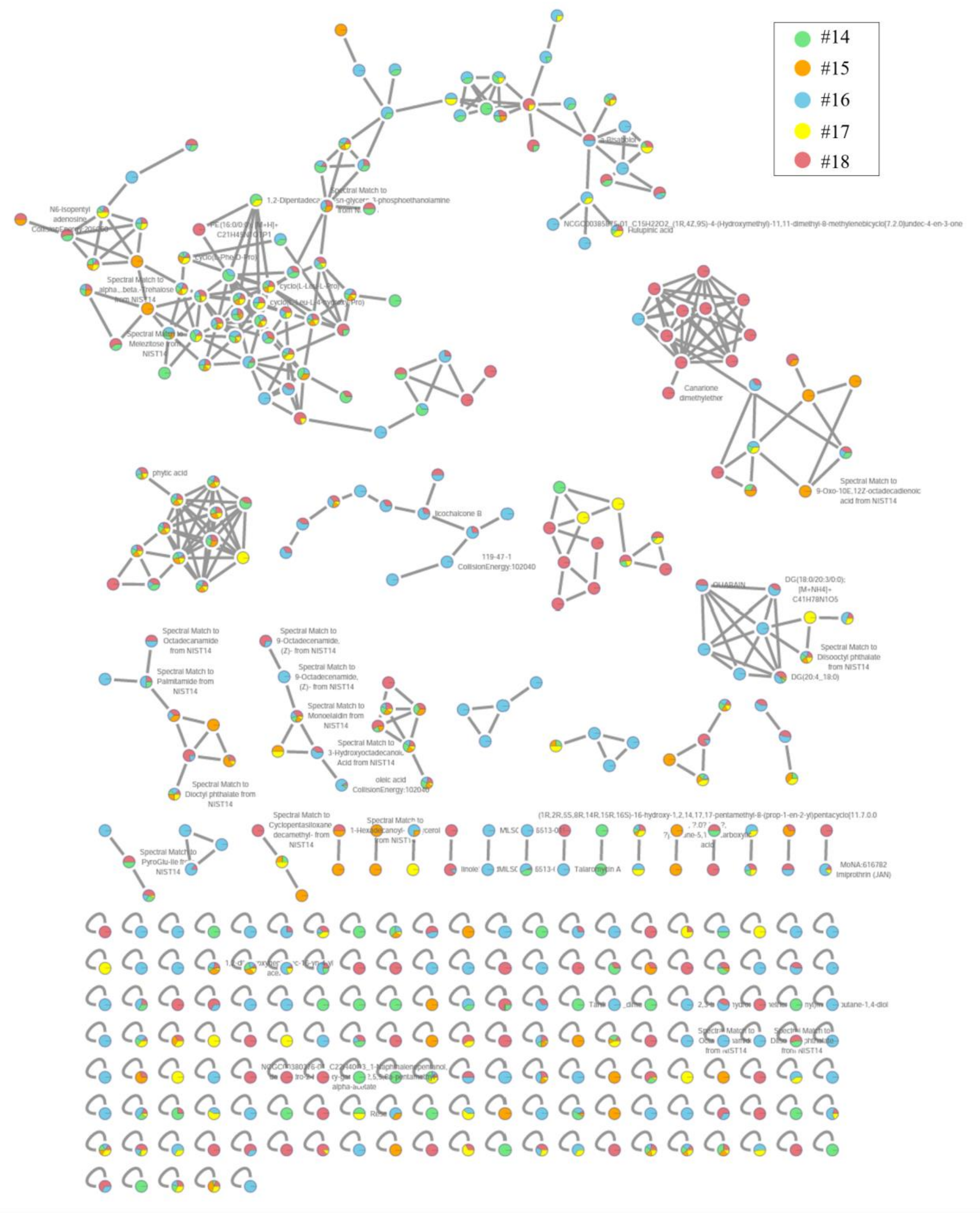

Figure S10. Molecular network of nutrient-rich liquid medium test

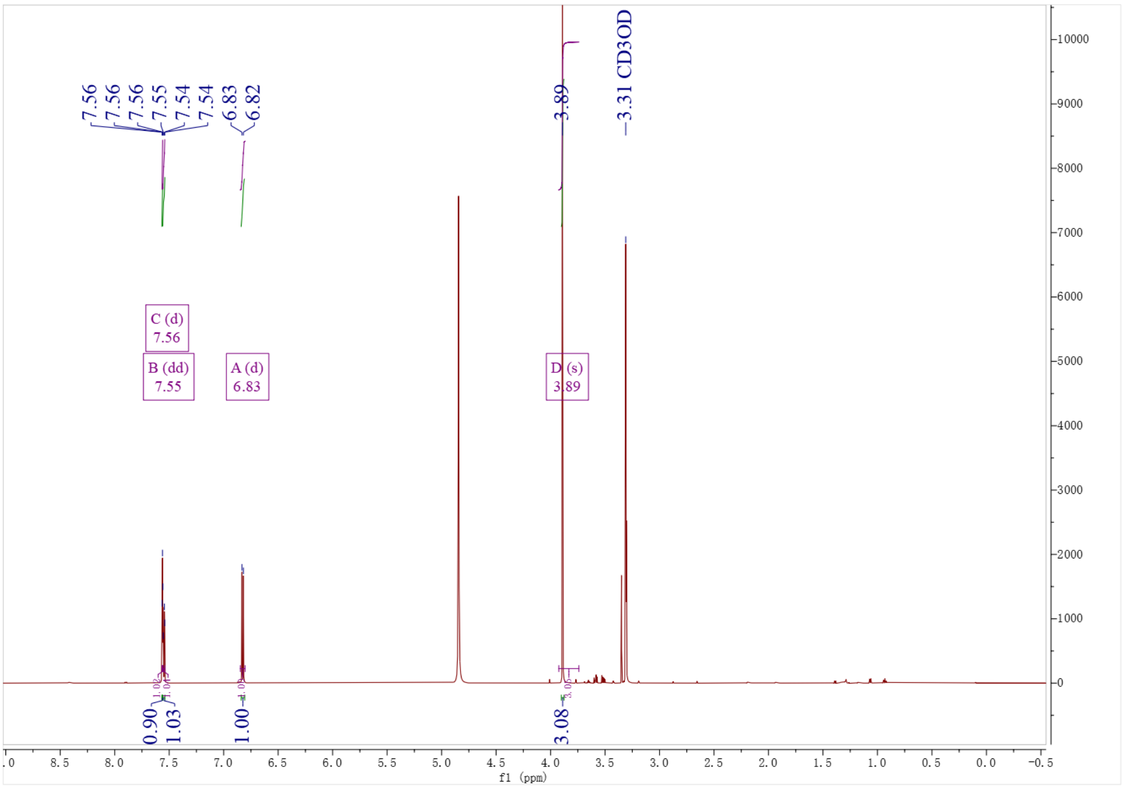

Figure S11. ESI-MS spectrum of compound **1**

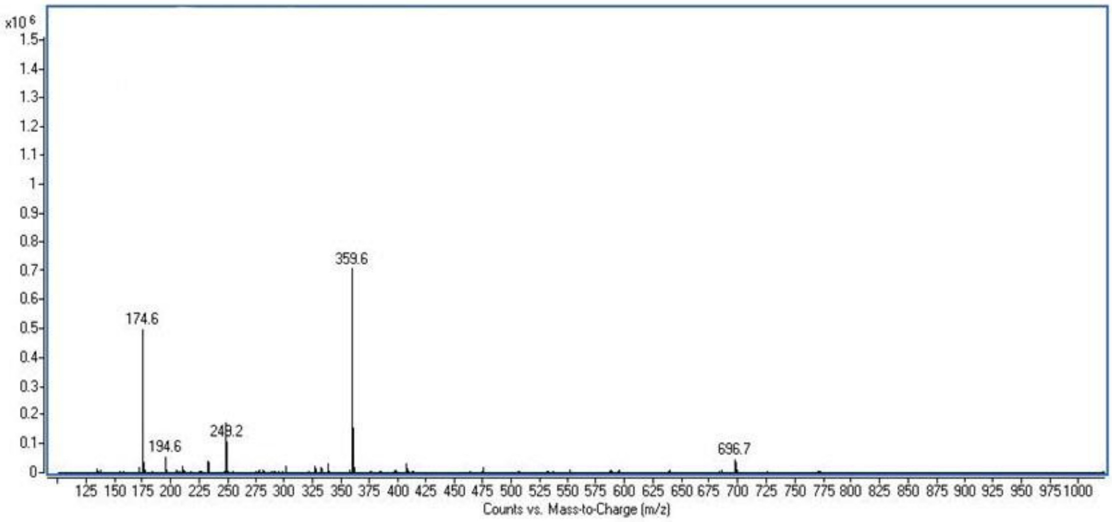

Figure S12. ^{1}H-NMR spectrum of compound **1** (CD_3OD, 600 MHz)

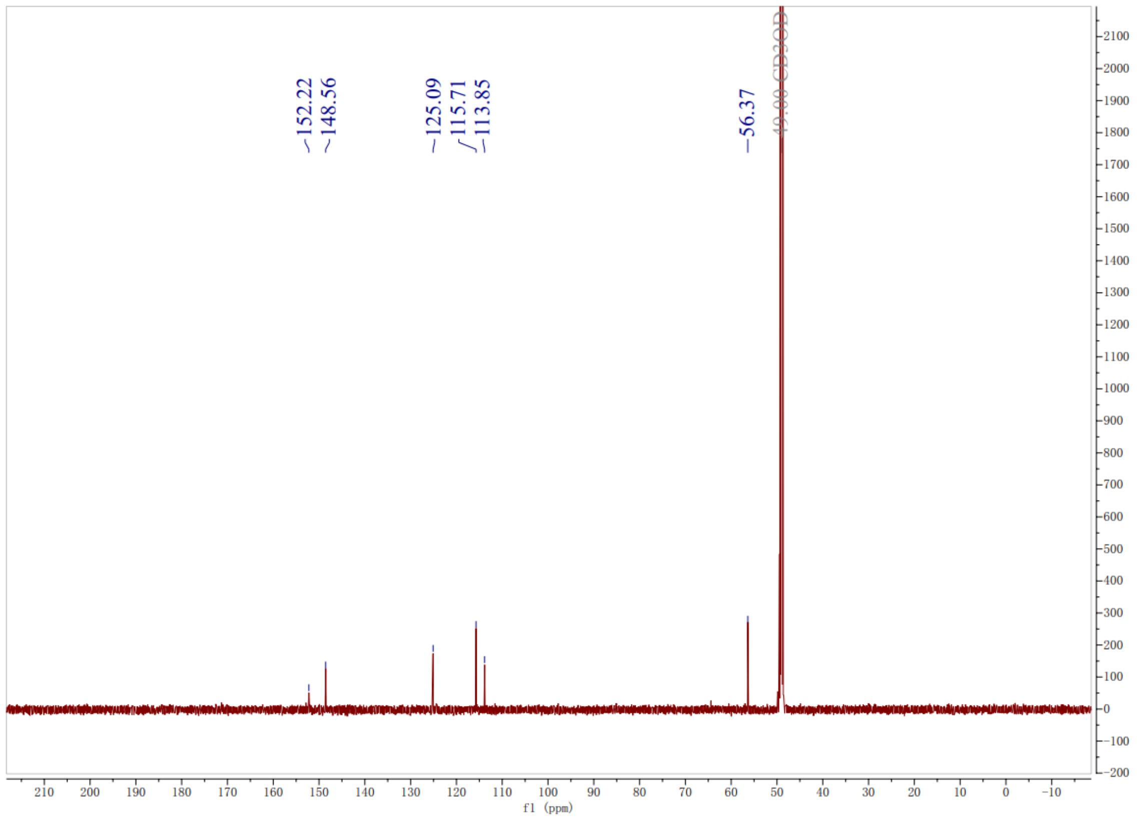

Figure S13. ^{13}C-NMR spectrum of compound **1**(CD_3OD, 150 MHz)

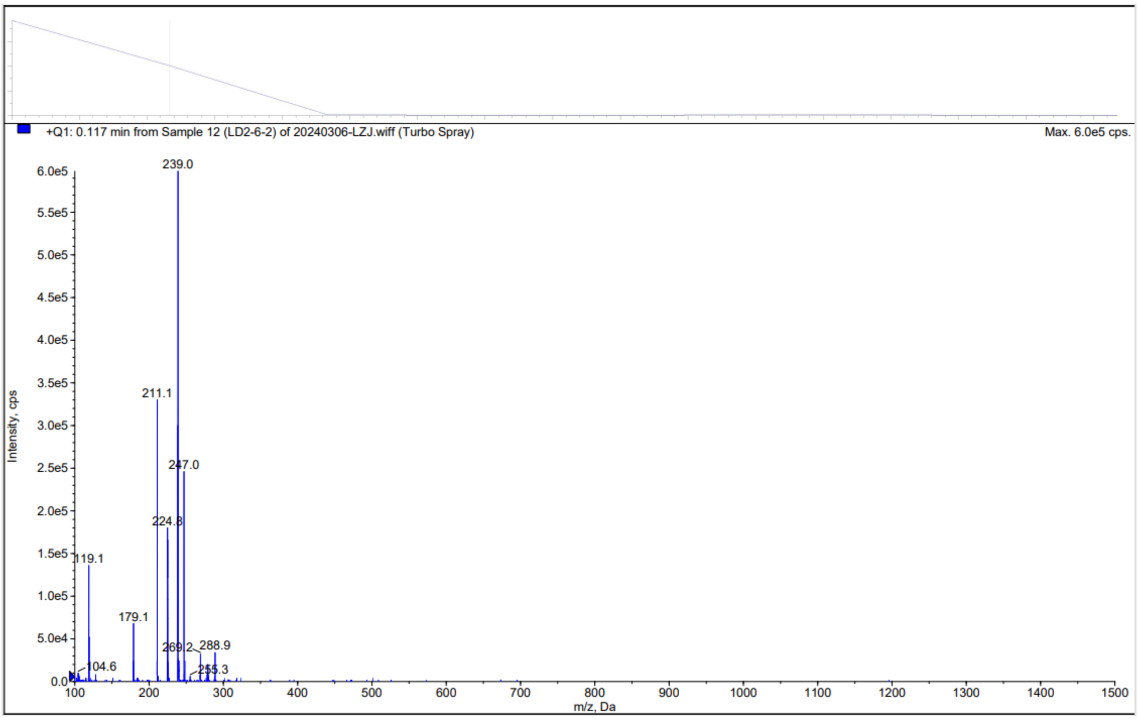

Figure S14. ESI-MS spectrum of compound **2**

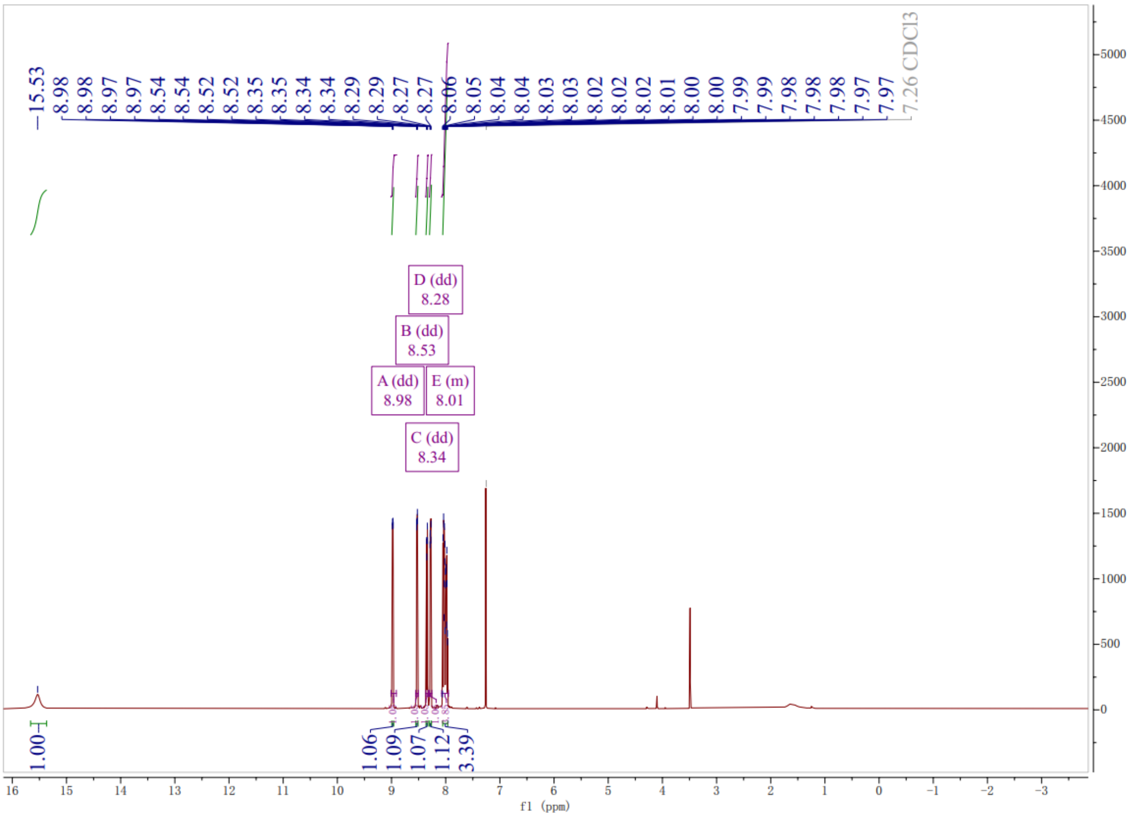

Figure S15. ^{1}H-NMR spectrum of compound **2** ($CDCl_3$, 600 MHz)

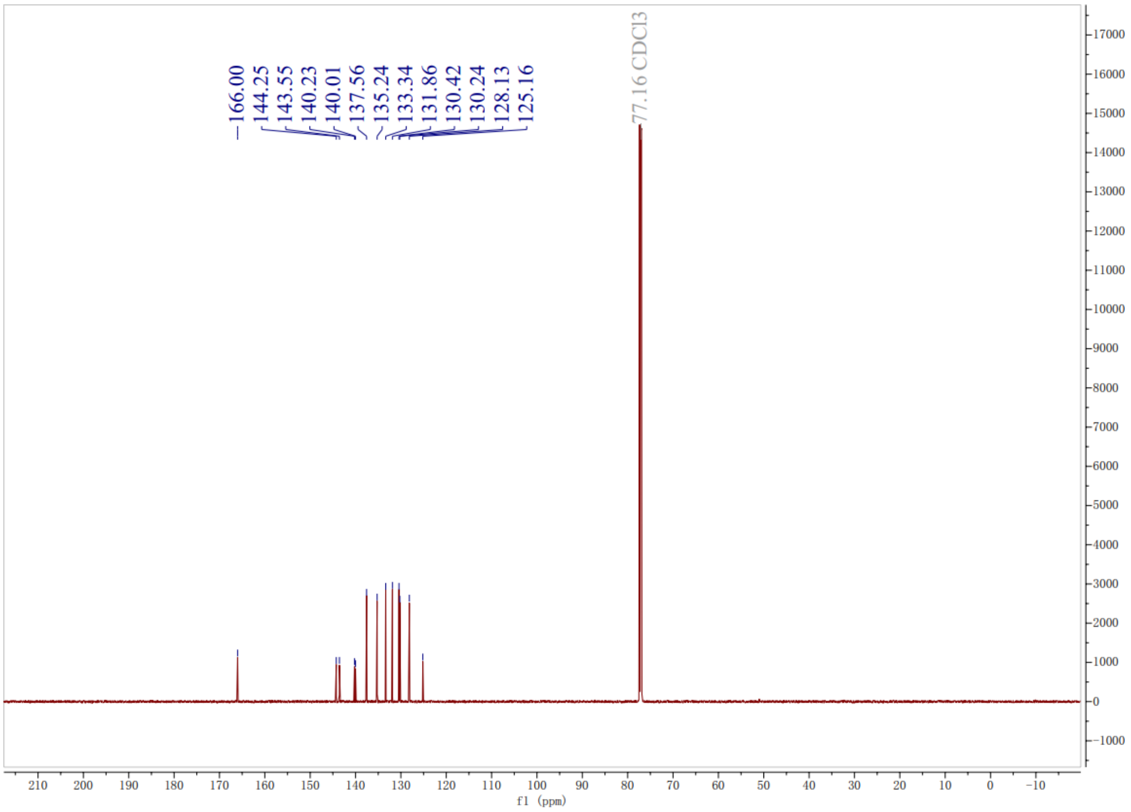

Figure S16. ^{13}C-NMR spectrum of compound **2** ($CDCl_3$, 150 MHz)

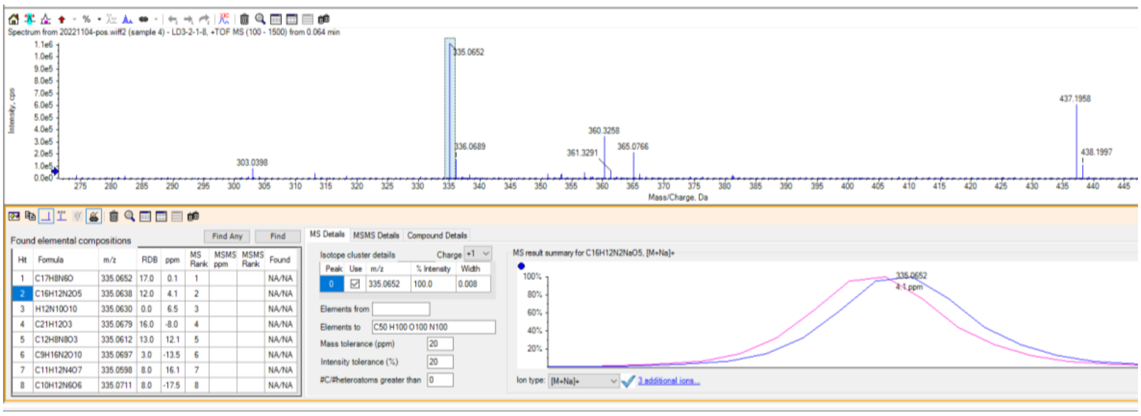

Figure S17. HRESI-MS spectrum of compound **3**

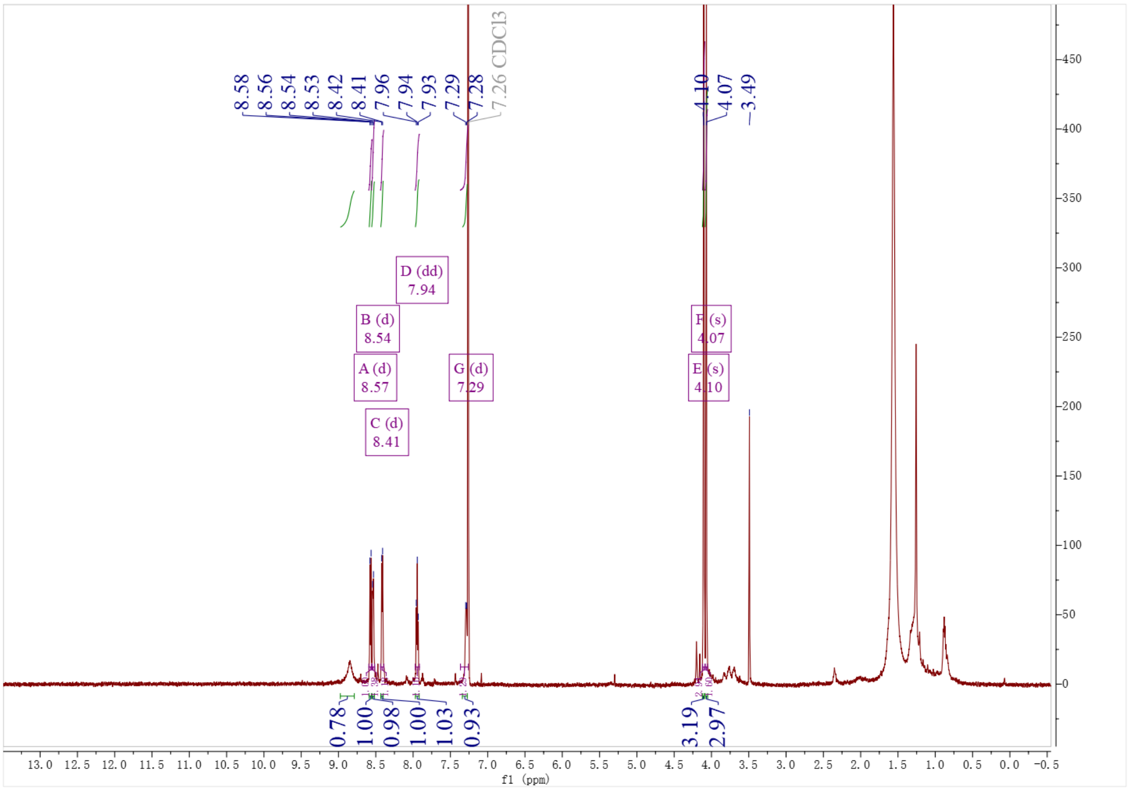

Figure S18. ^{1}H-NMR spectrum of compound **3** ($CDCl_3$, 600 MHz)

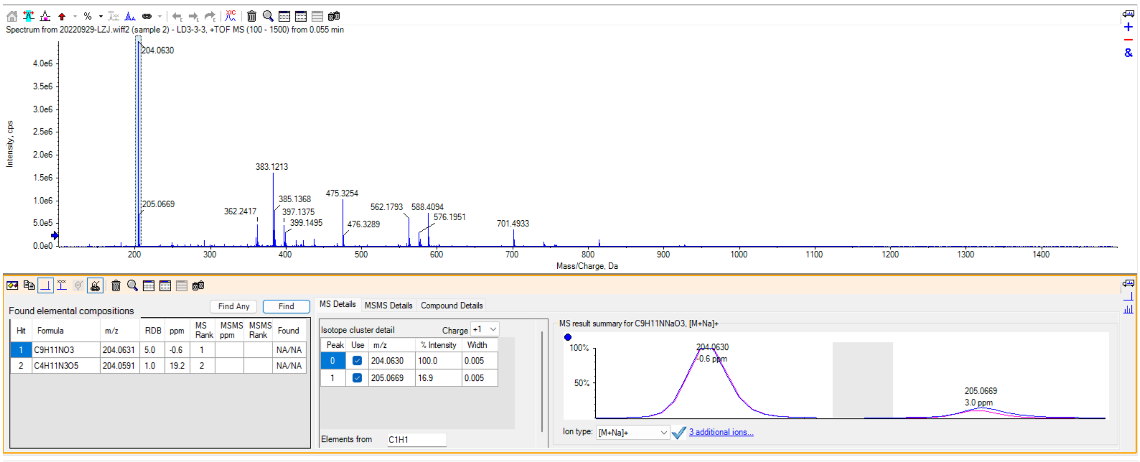

Figure S19. HRESI-MS spectrum of compound **4**

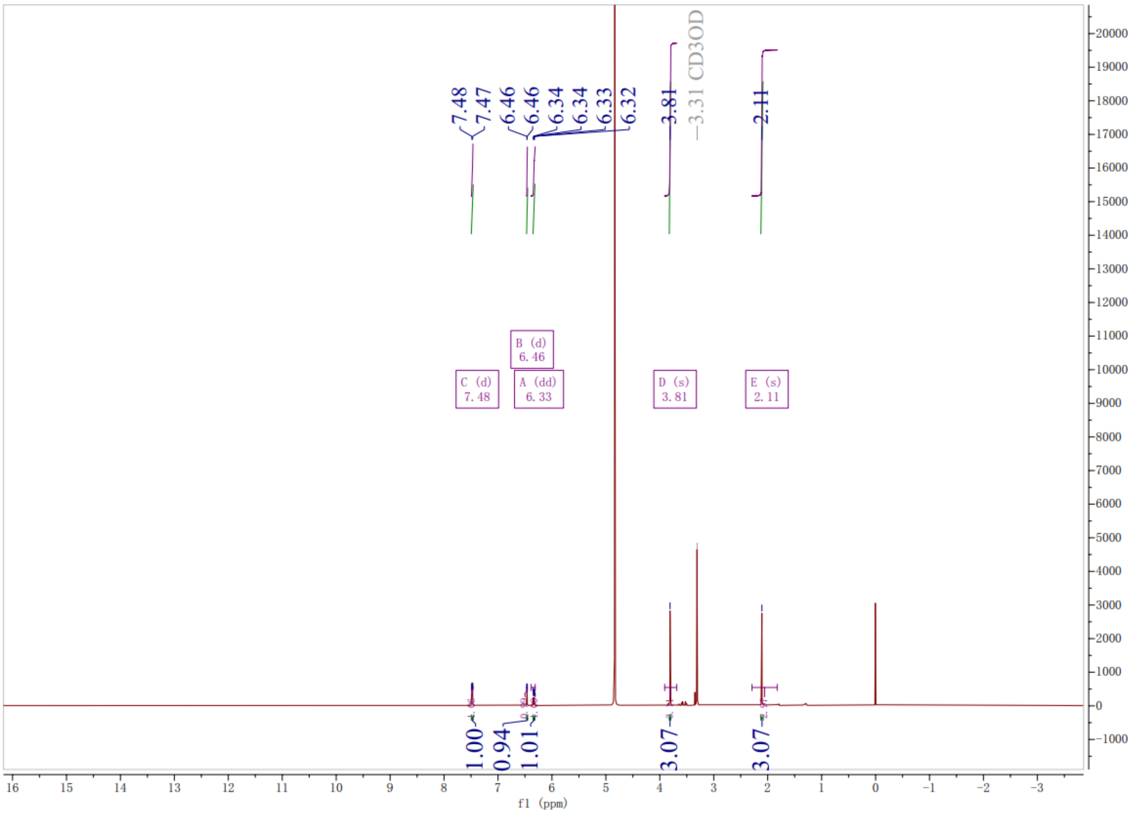

Figure S20. ^{1}H-NMR spectrum of compound **4** (CD_3OD, 600 MHz)

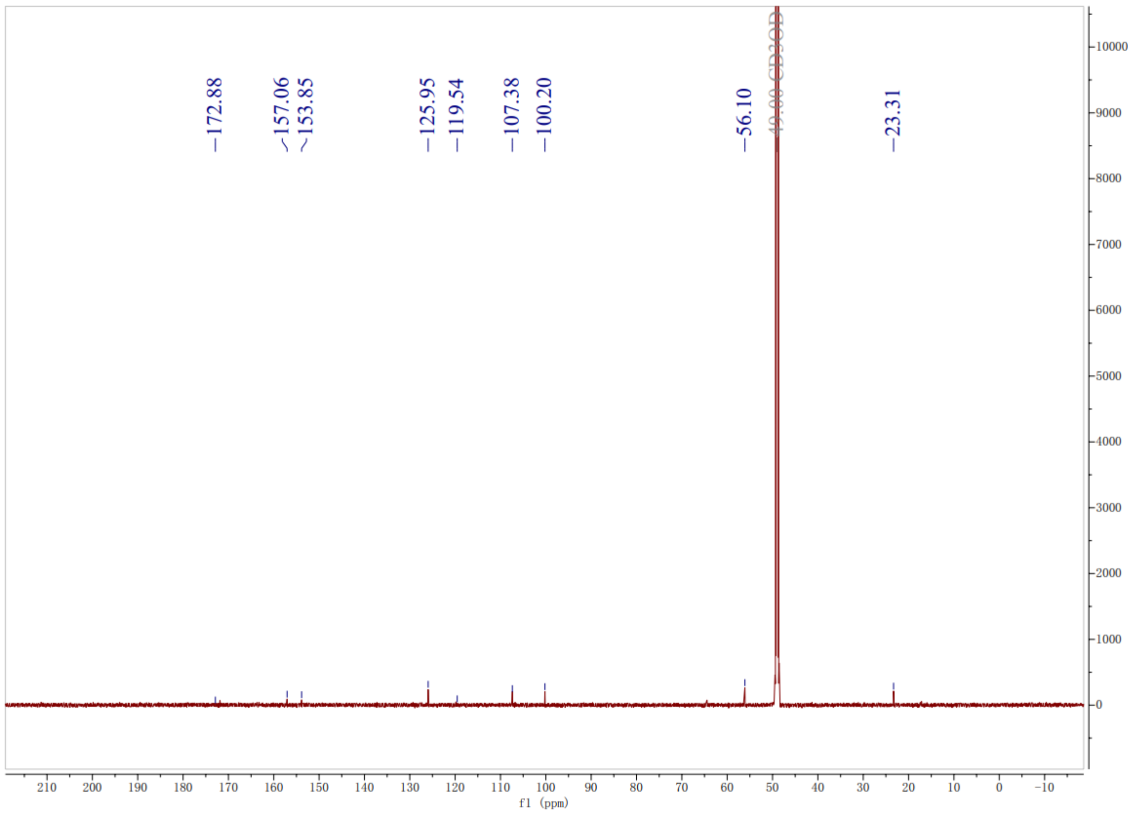

Figure S21. ^{13}C-NMR spectrum of compound **4** (CD_3OD, 150 MHz)

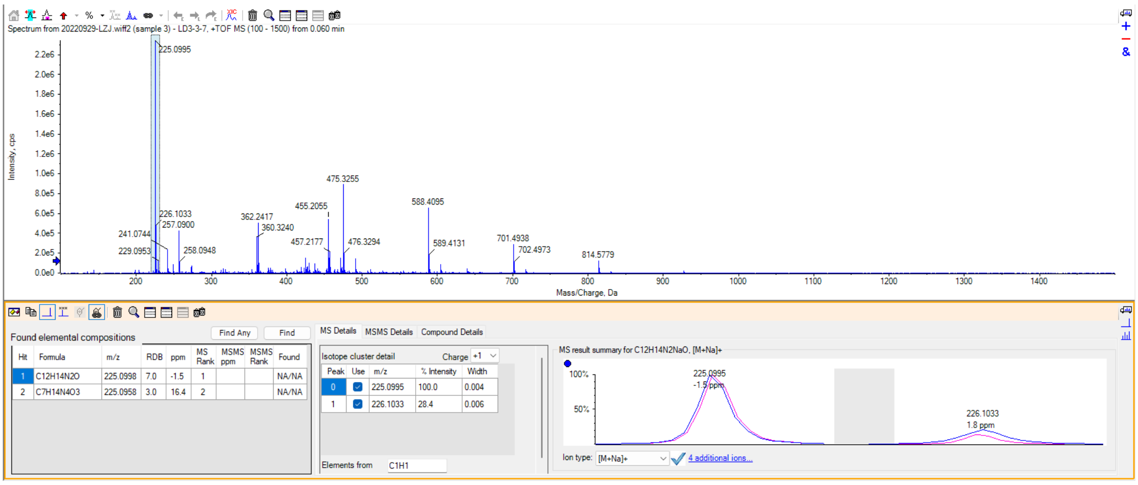

Figure S22. HRESI-MS spectrum of compound **5**

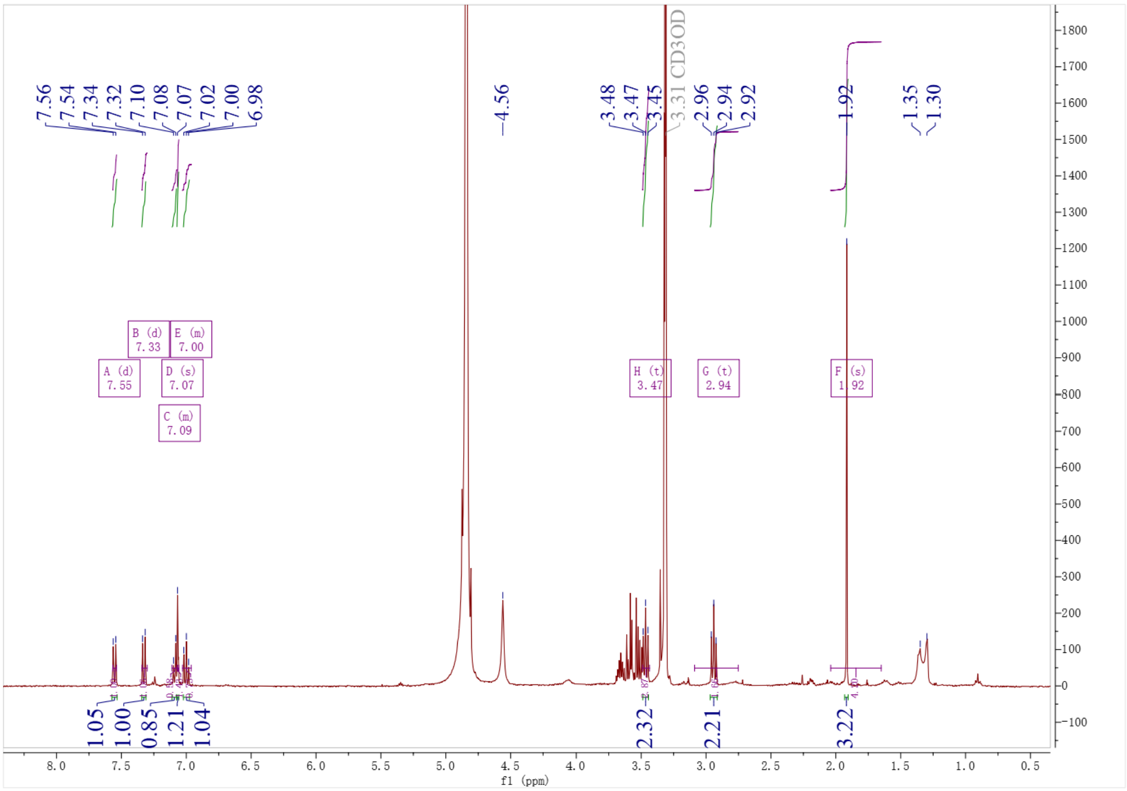

Figure S23. ^{1}H-NMR spectrum of compound **5** (CD_3OD, 600 MHz)

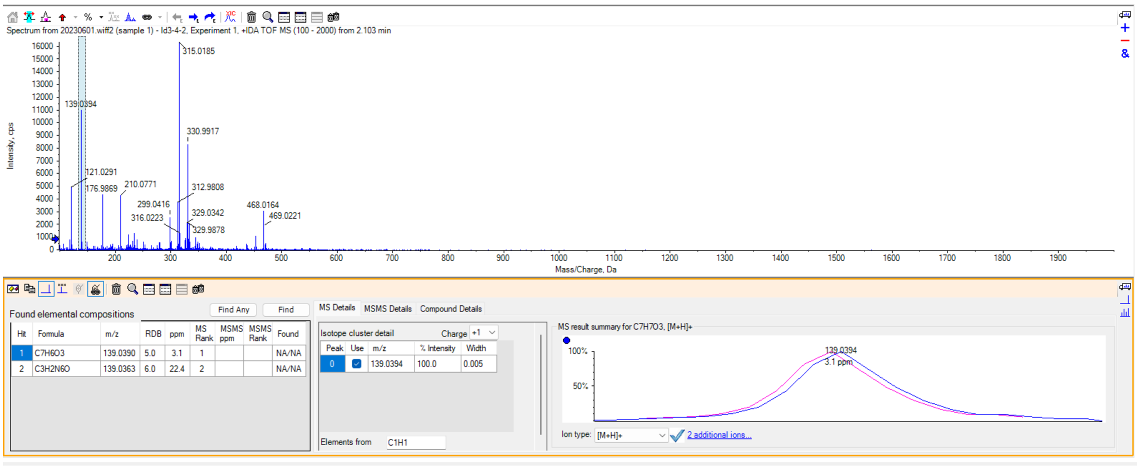

Figure S24. HRESI-MS spectrum of compound **6**

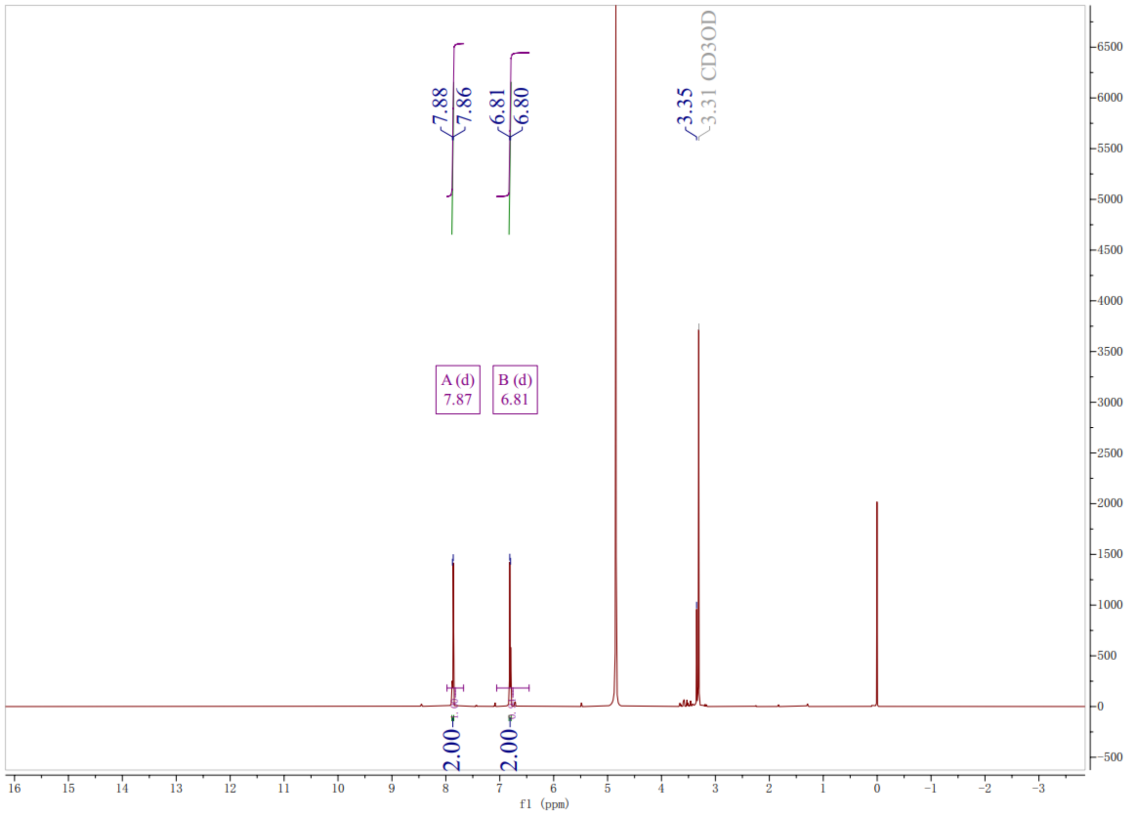

Figure S25. ^{1}H-NMR spectrum of compound **6** (CD_3OD, 600 MHz)

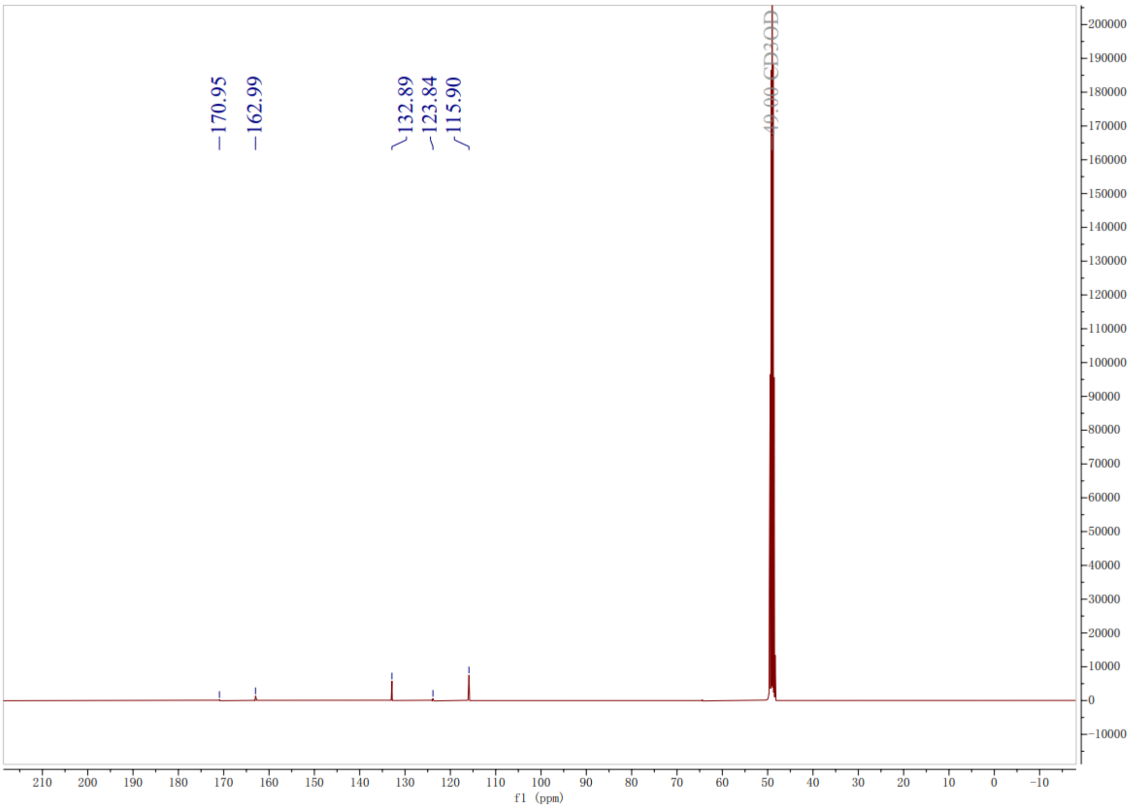

Figure S26. ^{13}C-NMR spectrum of compound **6** (CD_3OD, 150 MHz)

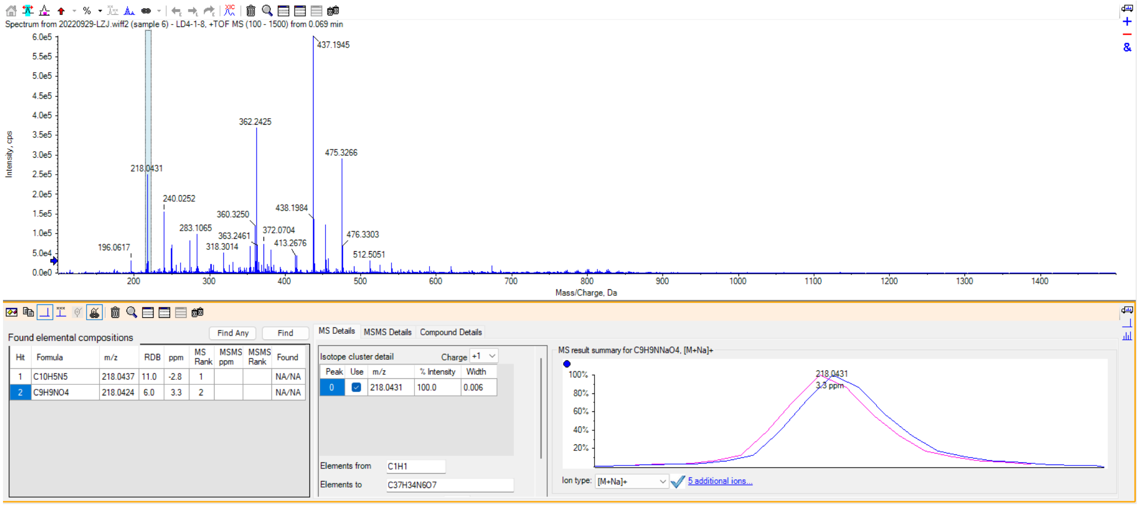

Figure S27. HRESI-MS spectrum of compound **7**

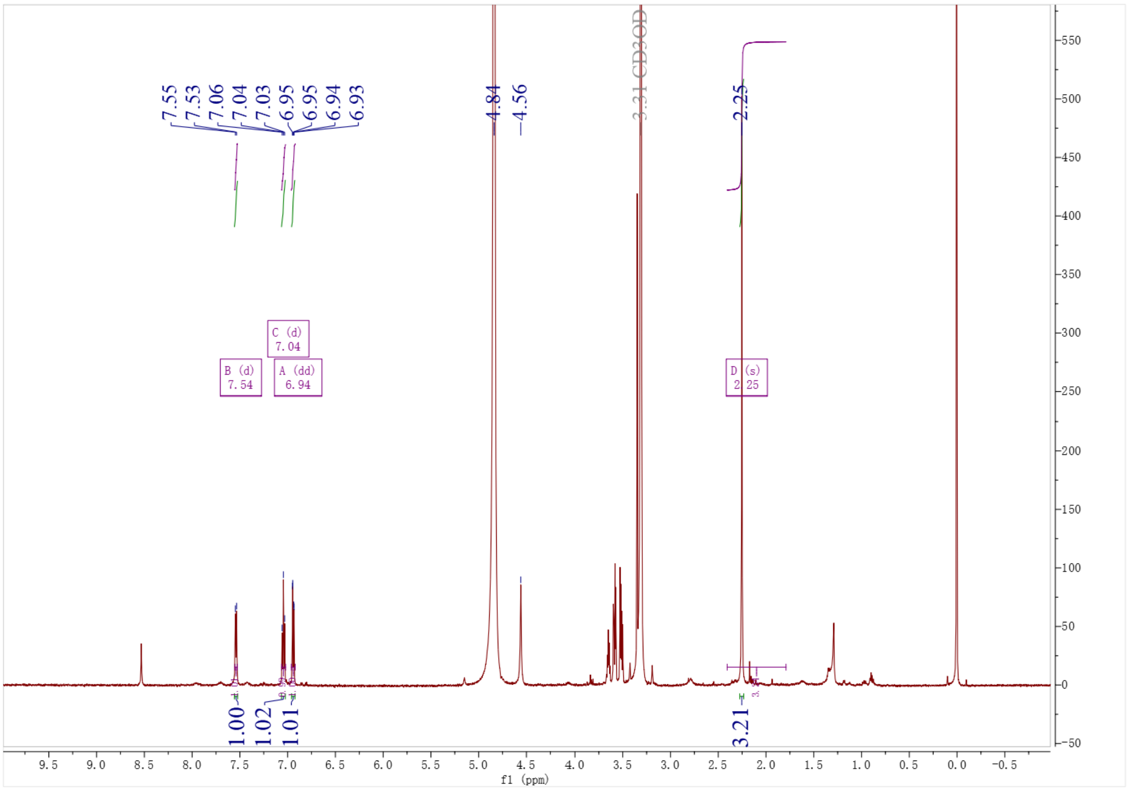

Figure S28. ^{1}H-NMR spectrum of compound **7** (CD_3OD, 600 MHz)

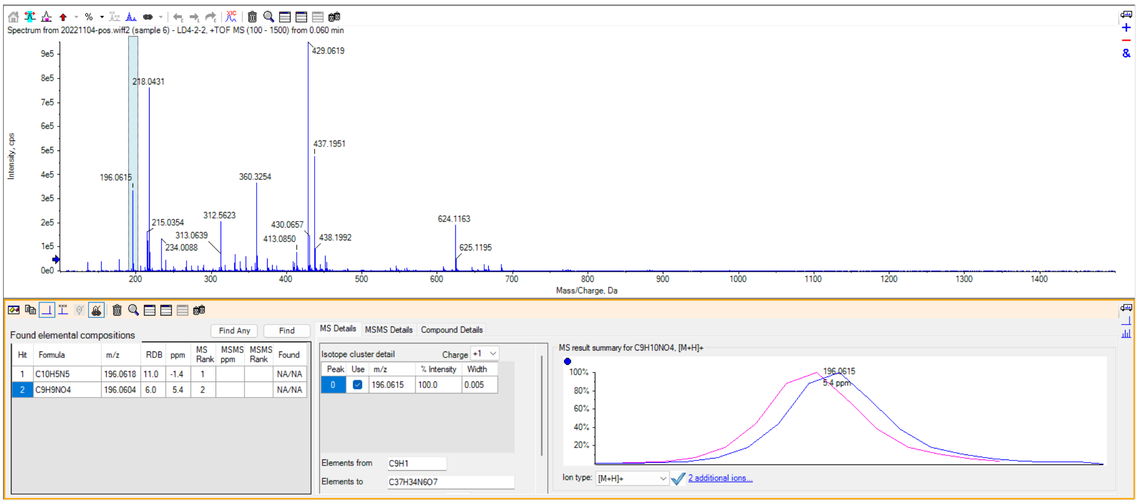

Figure S29. HRESI-MS spectrum of compound **8**

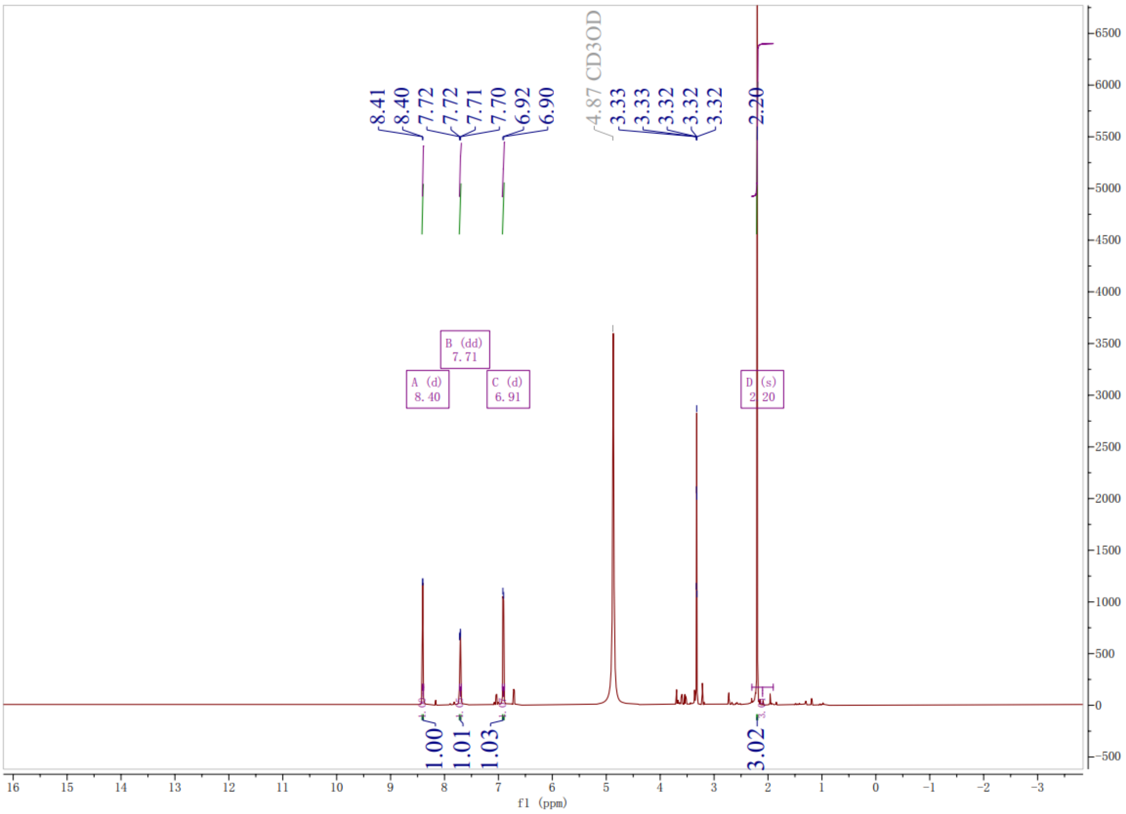

Figure S30. ^{1}H-NMR spectrum of compound **8** (CD_3OD, 600 MHz)

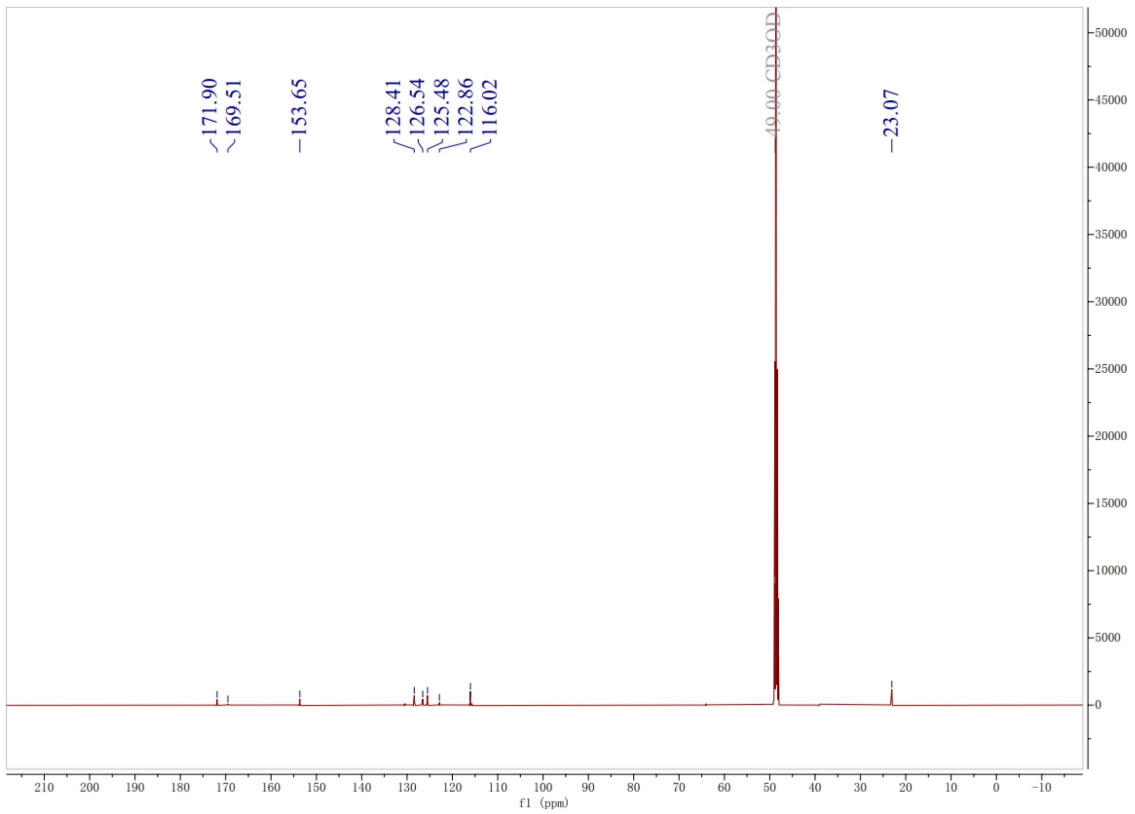

Figure S31. ^{13}C-NMR spectrum of compound **8** (CD_3OD, 150 MHz)

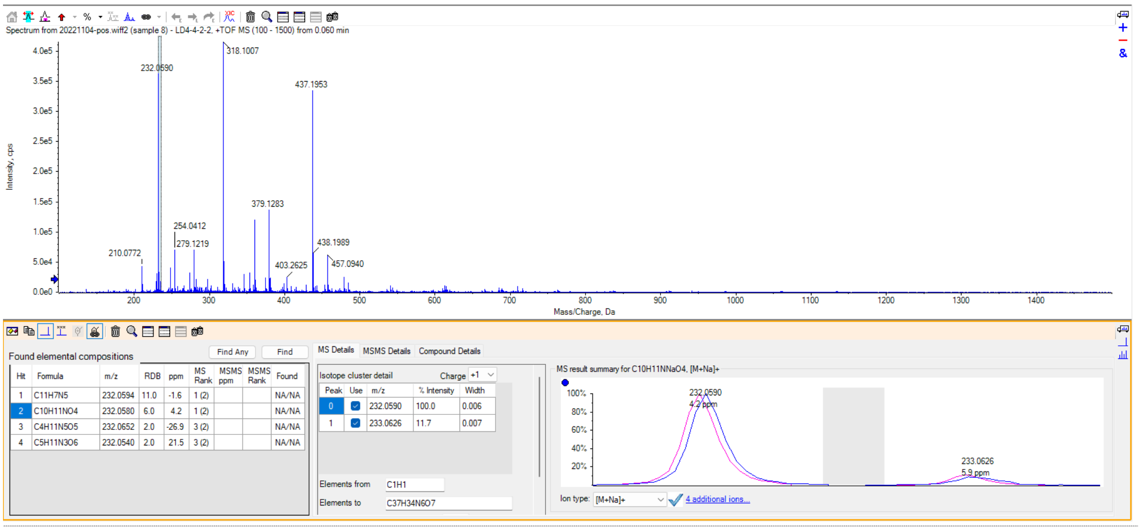

Figure S32. HRESI-MS spectrum of compound **9**

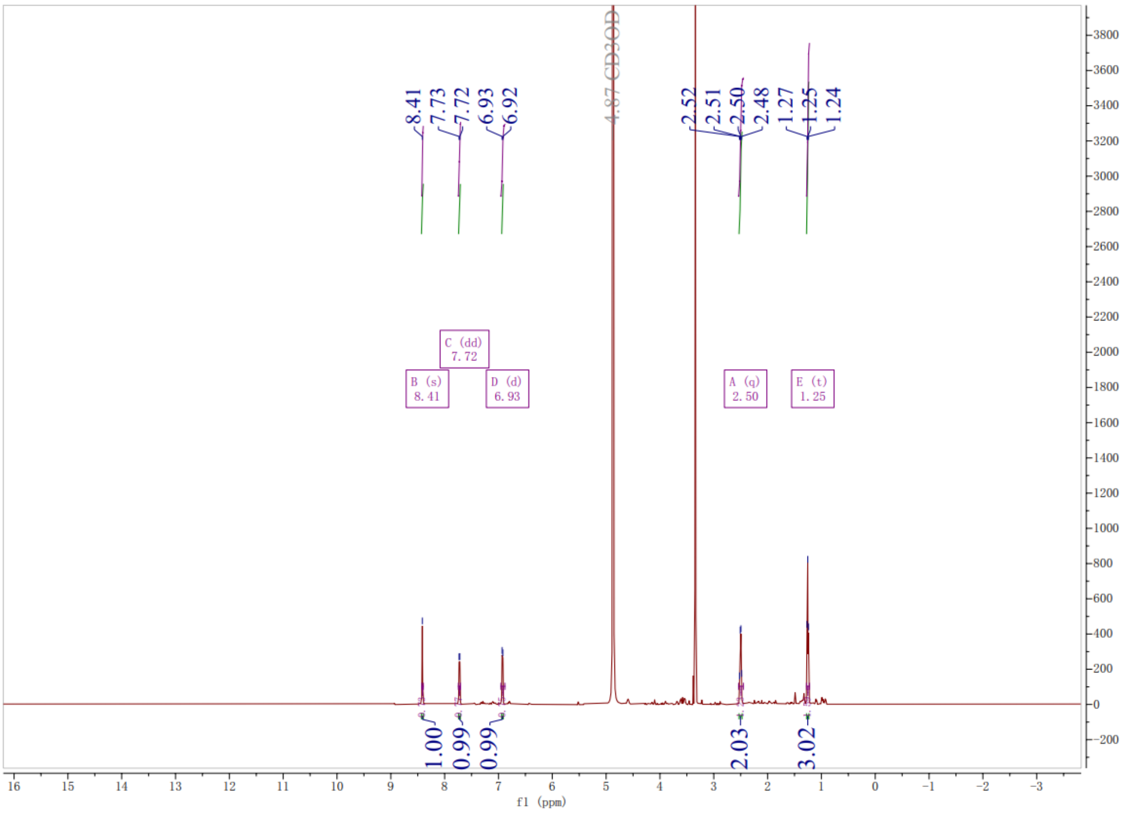

Figure S33. ^{1}H-NMR spectrum of compound **9** (CD_3OD, 600 MHz)

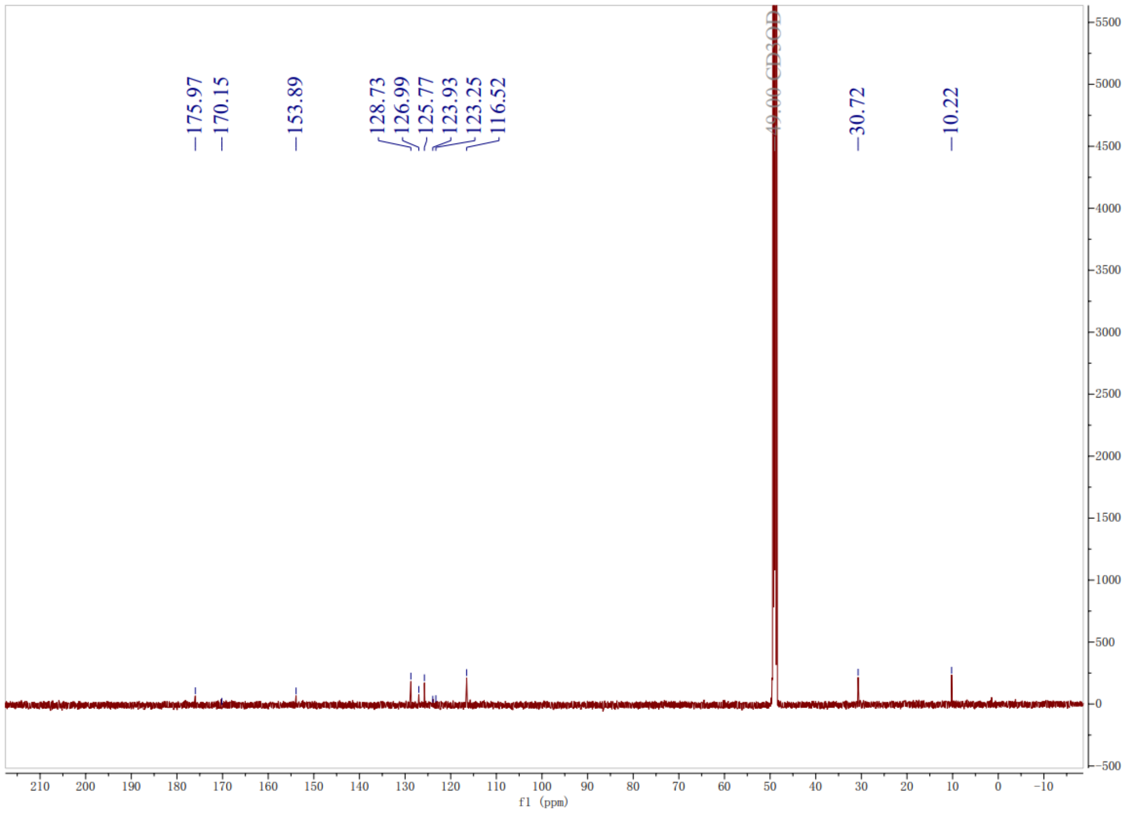

Figure S34. ^{13}C-NMR spectrum of compound **9** (CD_3OD, 150 MHz)

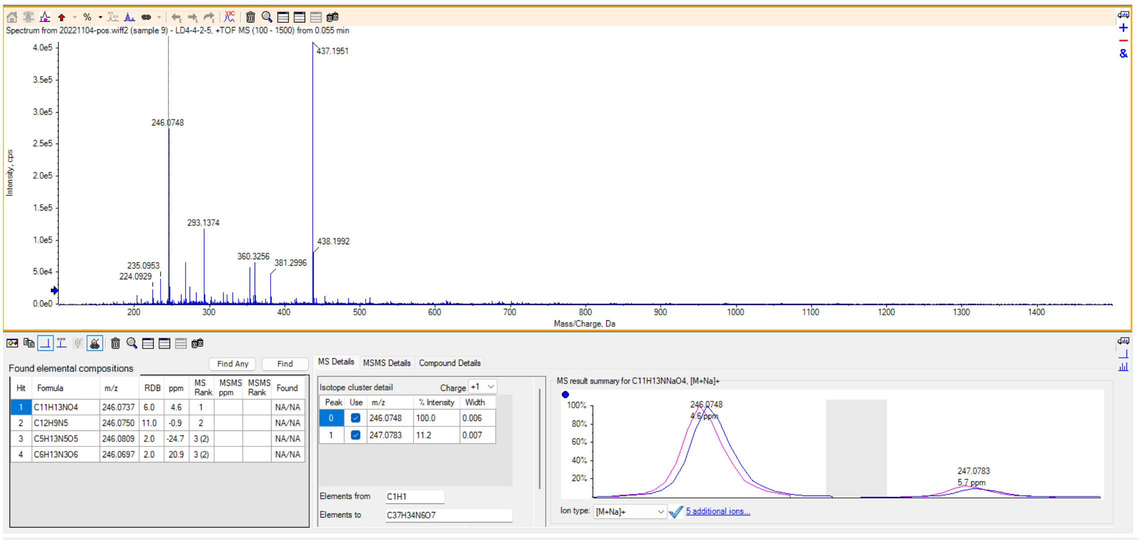

Figure S35. HRESI-MS spectrum of compound **10**

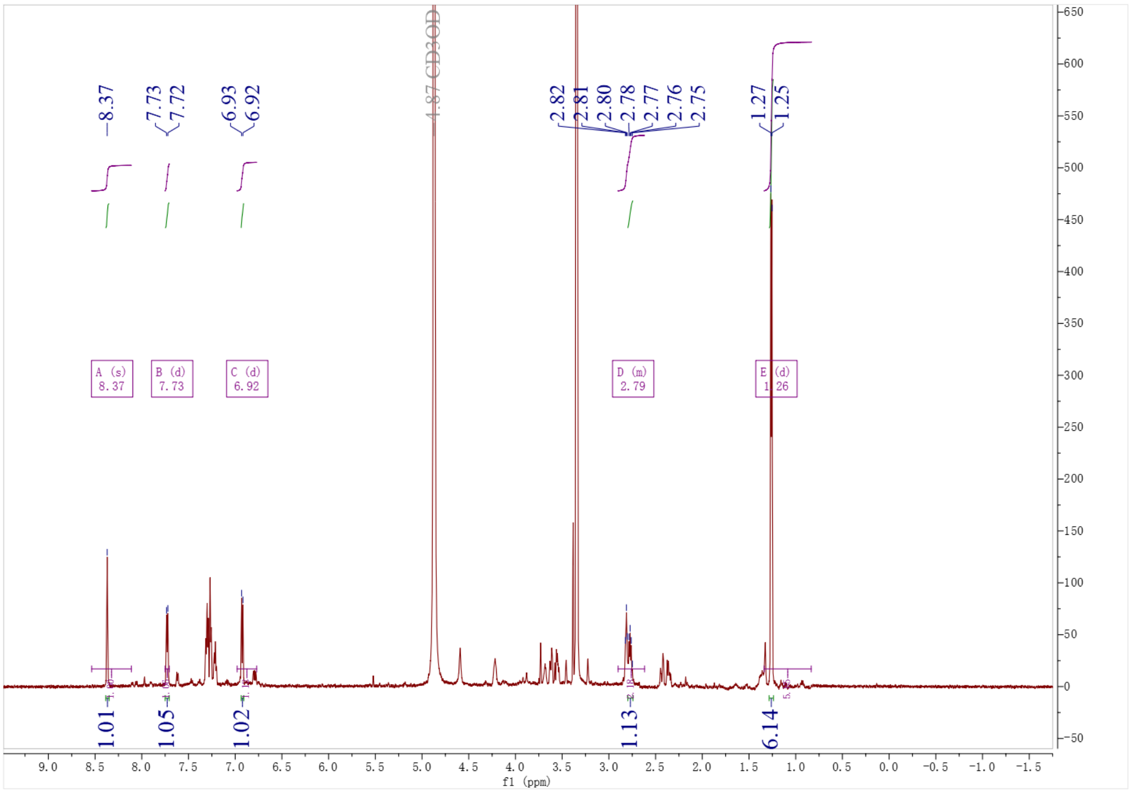

Figure S36. ^{1}H-NMR spectrum of compound **10** (CD_3OD, 600 MHz)

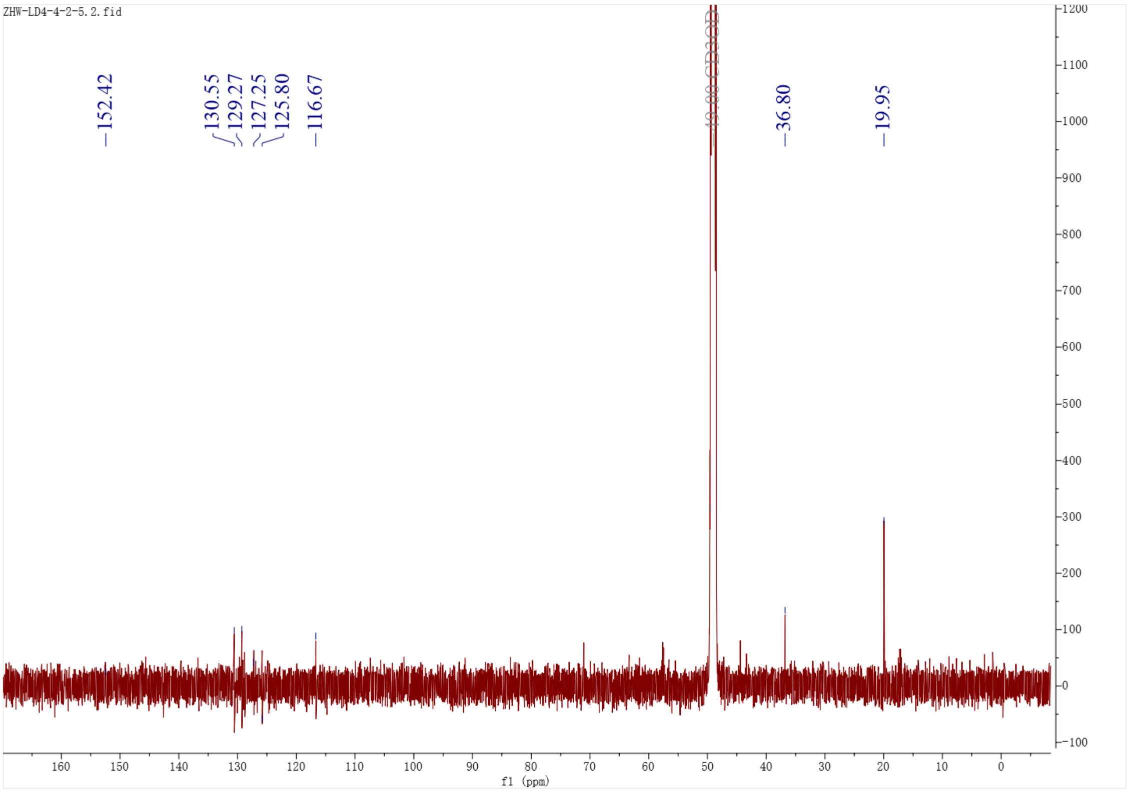

Figure S37. ^{13}C-NMR spectrum of compound **10** (CD_3OD, 150 MHz)

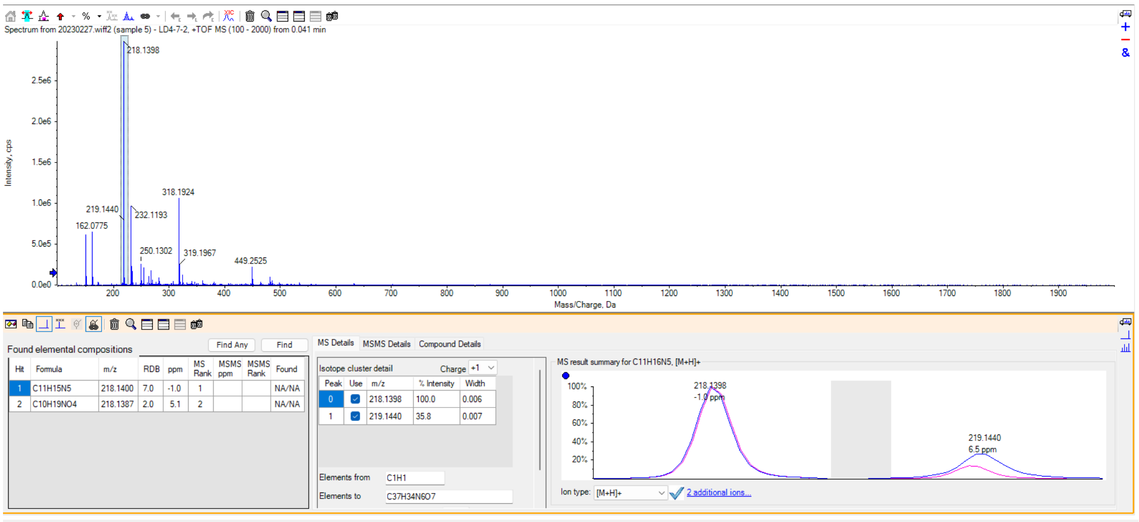

Figure S38. HRESI-MS spectrum of compound **11**

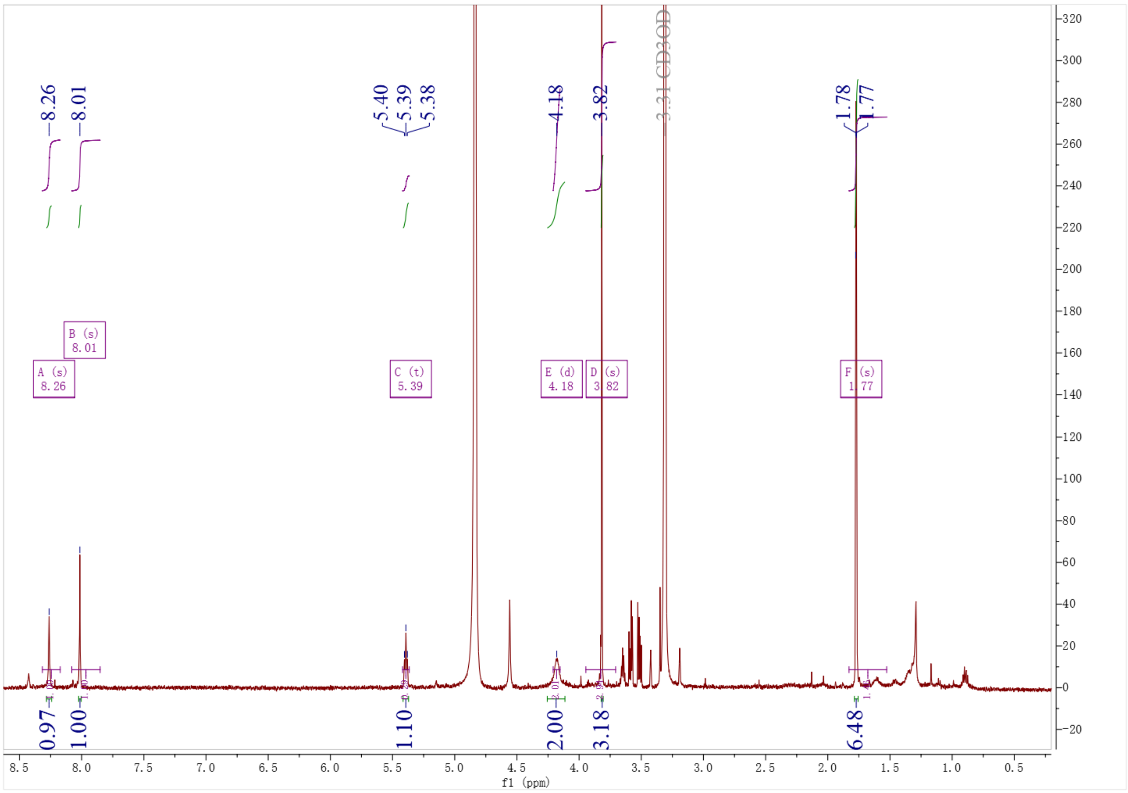

Figure S39. ^{1}H-NMR spectrum of compound **11** (CD_3OD, 600 MHz)

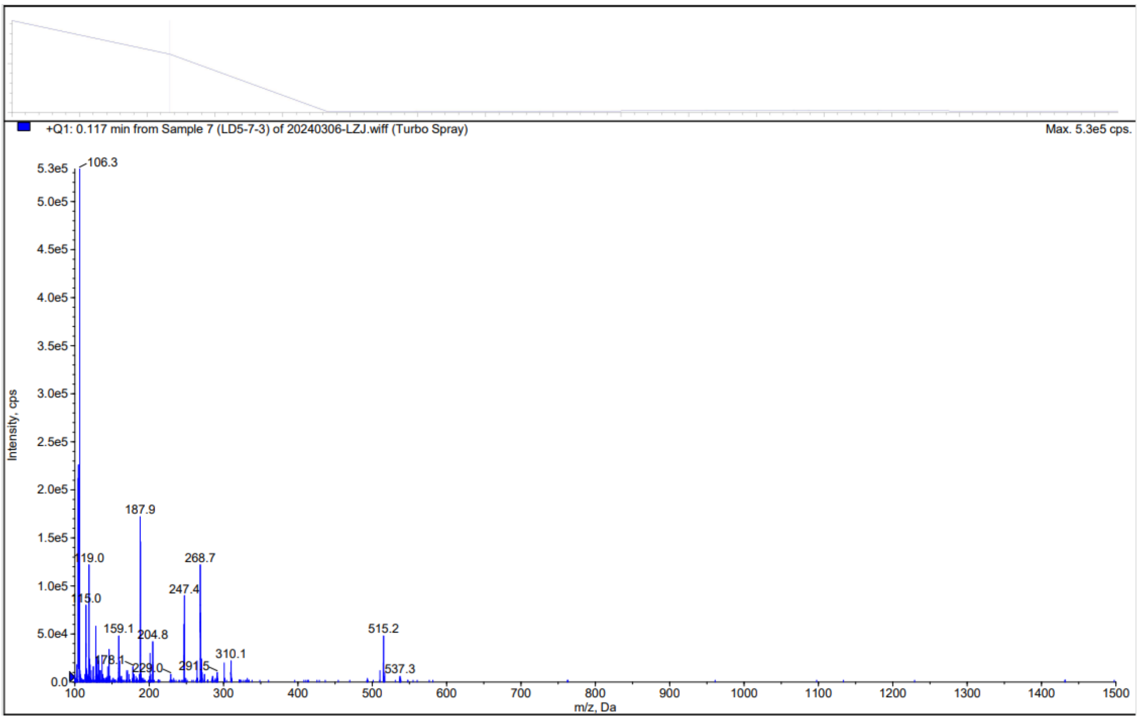

Figure S40. ESI-MS spectrum of compound **12**

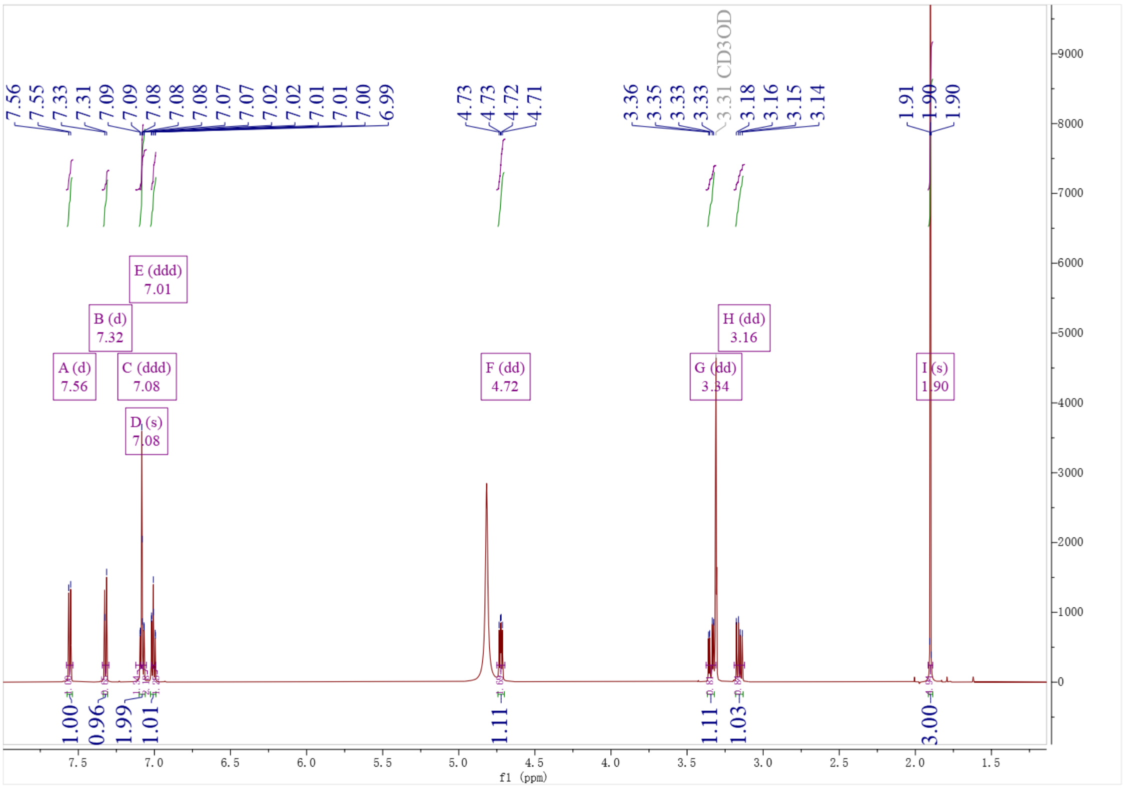

Figure S41. ^{1}H-NMR spectrum of compound **12** (CD_3OD, 600 MHz)

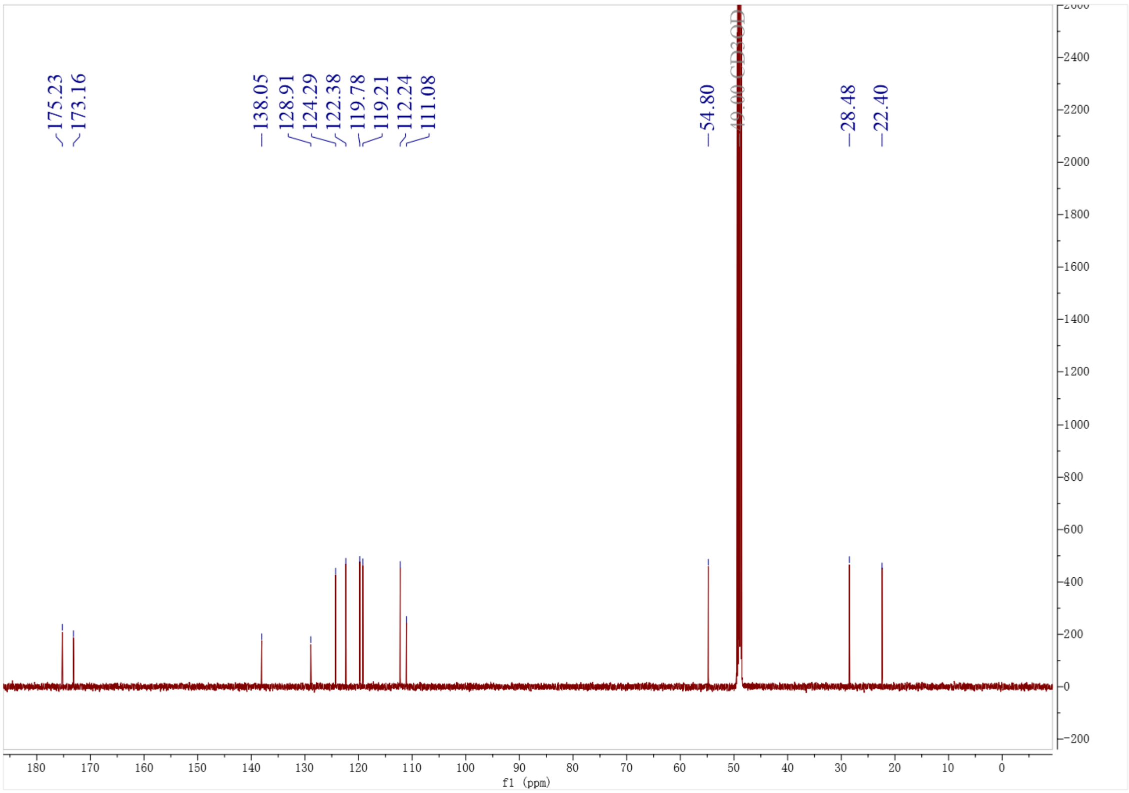

Figure S42. ^{13}C-NMR spectrum of compound **12** (CD_3OD, 150 MHz)

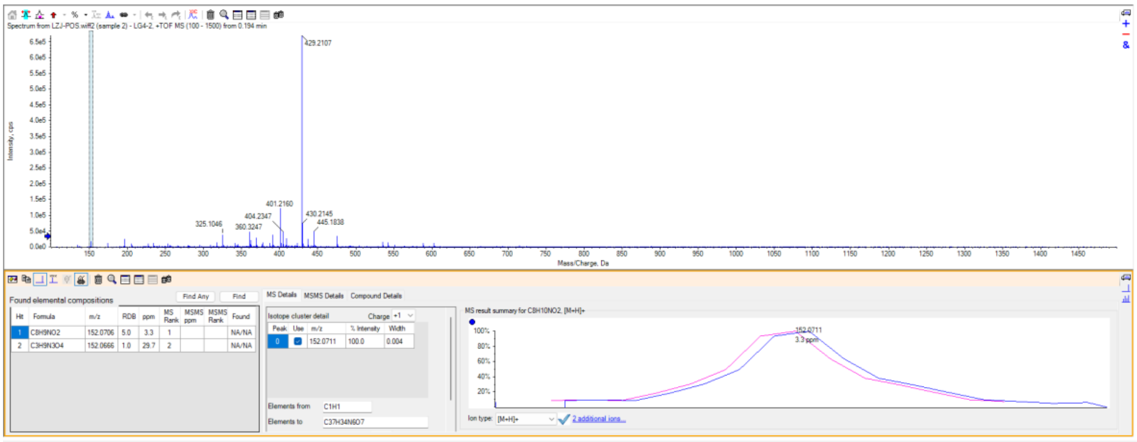

Figure S43. HRESI-MS spectrum of compound **13**

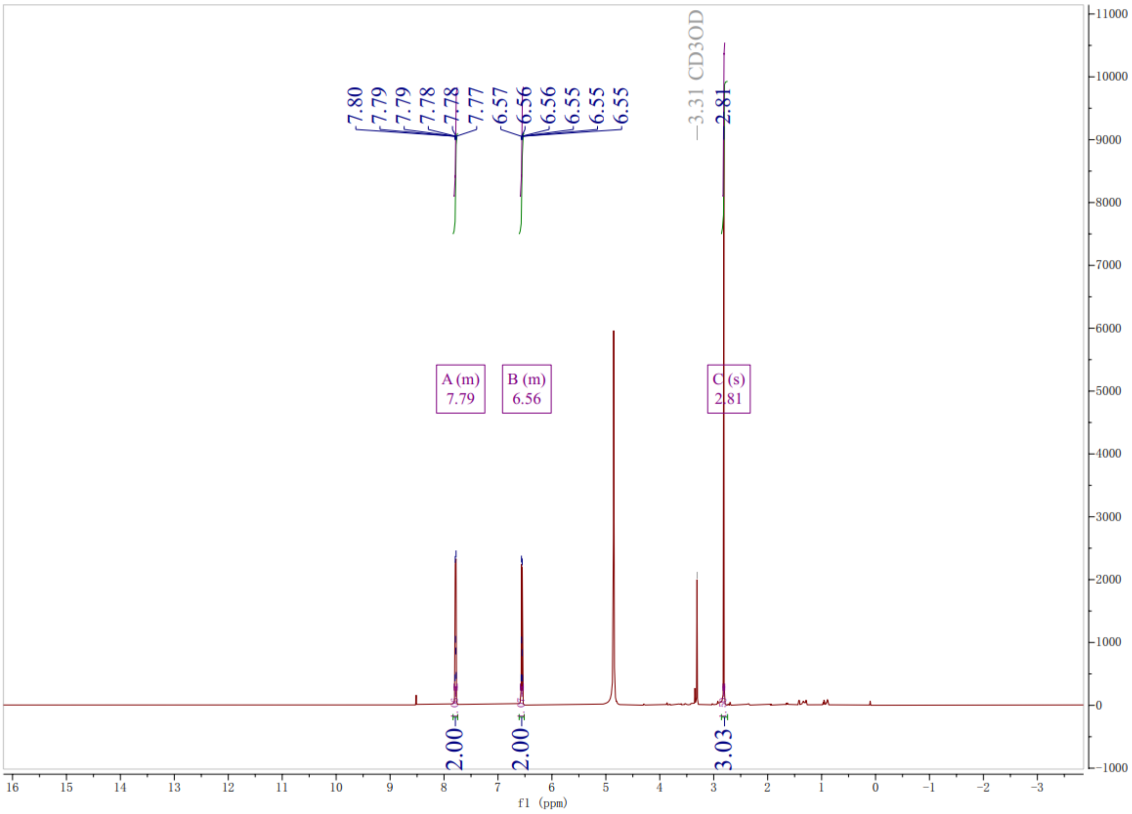

Figure S44. ^{1}H-NMR spectrum of compound **13** (CD_3OD, 600 MHz)

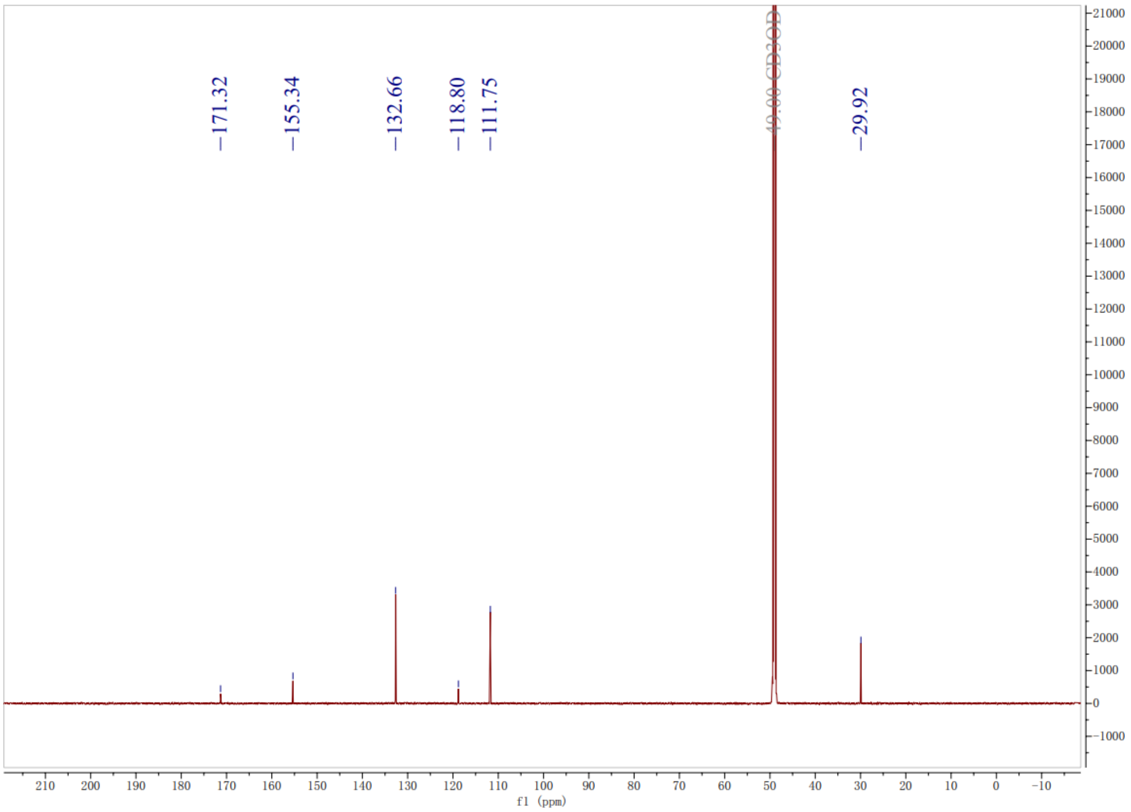

Figure S45. ^{13}C-NMR spectrum of compound **13** (CD_3OD, 150 MHz)

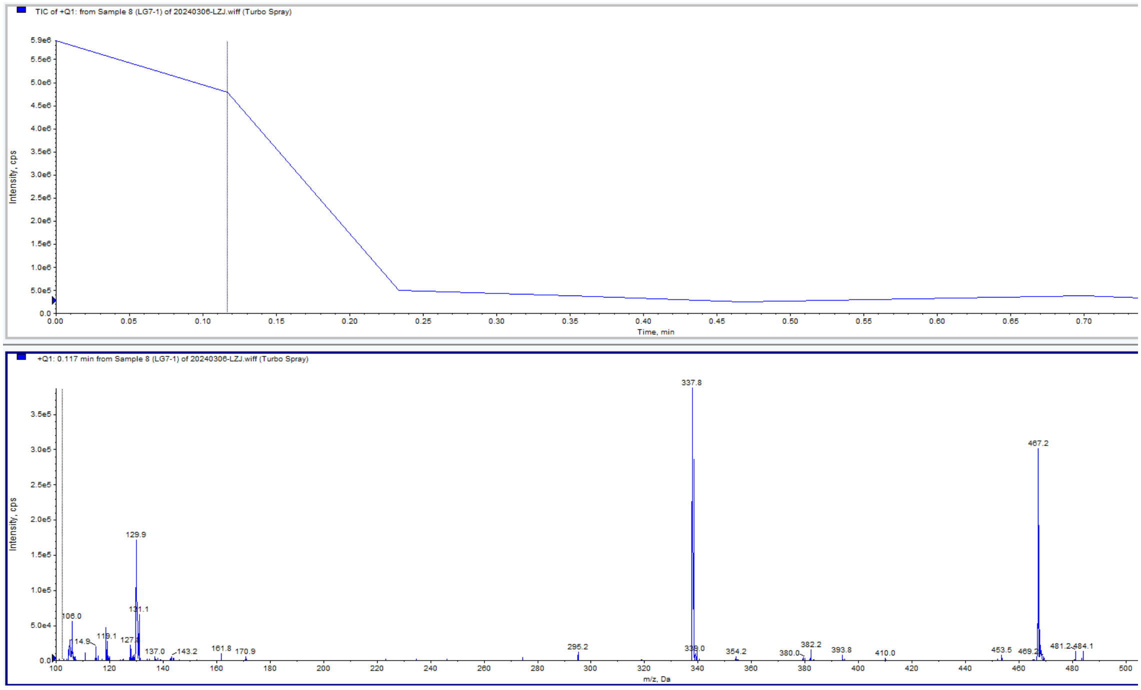

Figure S46. ESI-MS spectrum of compound **14**

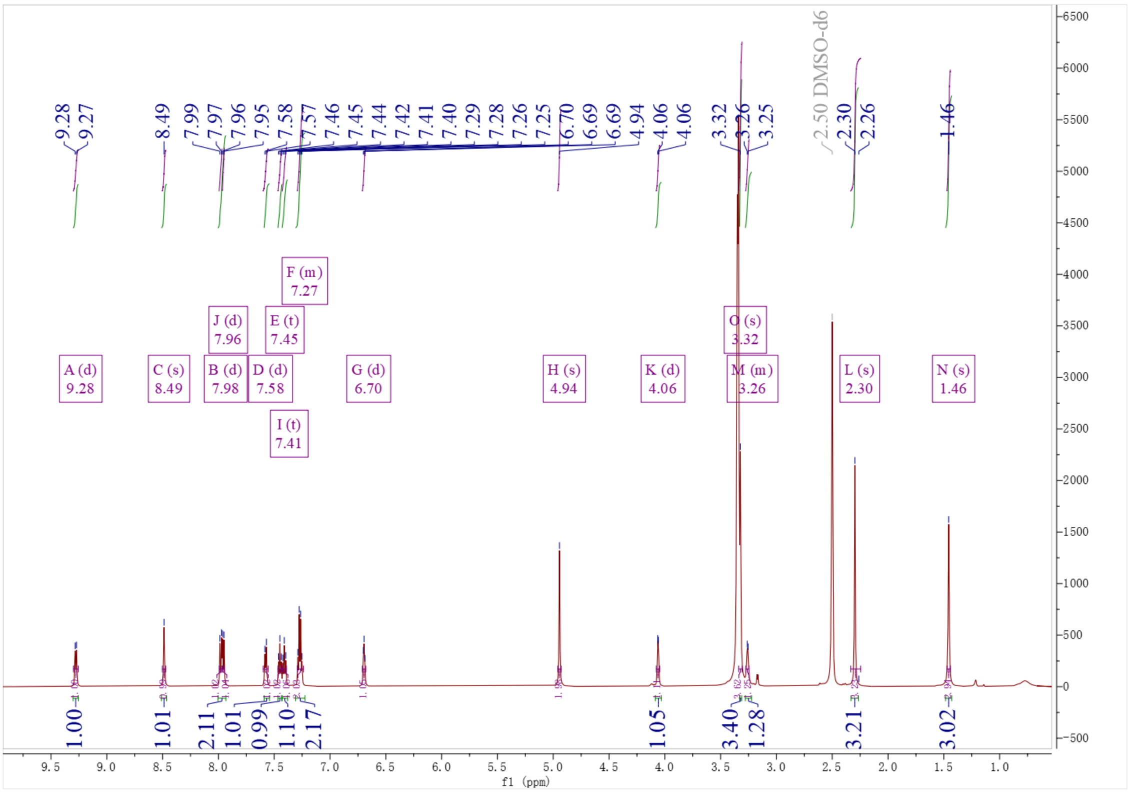

Figure S47. ^{1}H-NMR spectrum of compound **14** (DMSO-d6, 600 MHz)

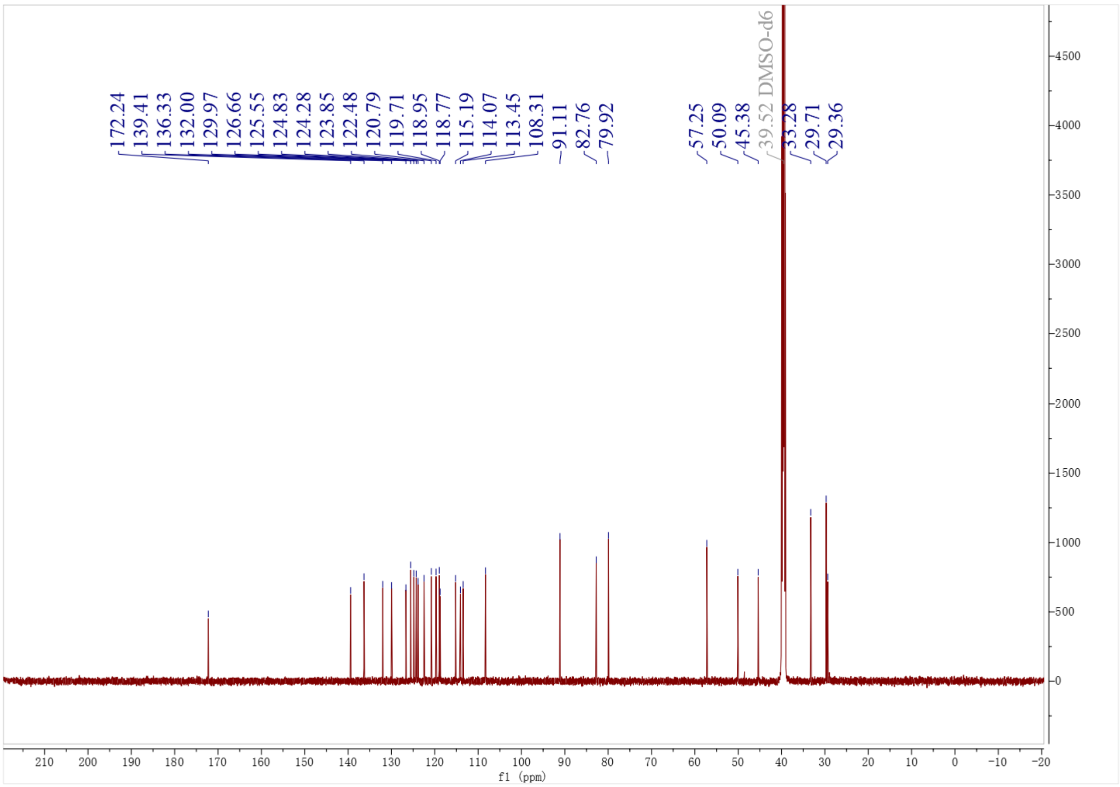

Figure S48. ^{13}C-NMR spectrum of compound **14** (DMSO-d6, 150 MHz)

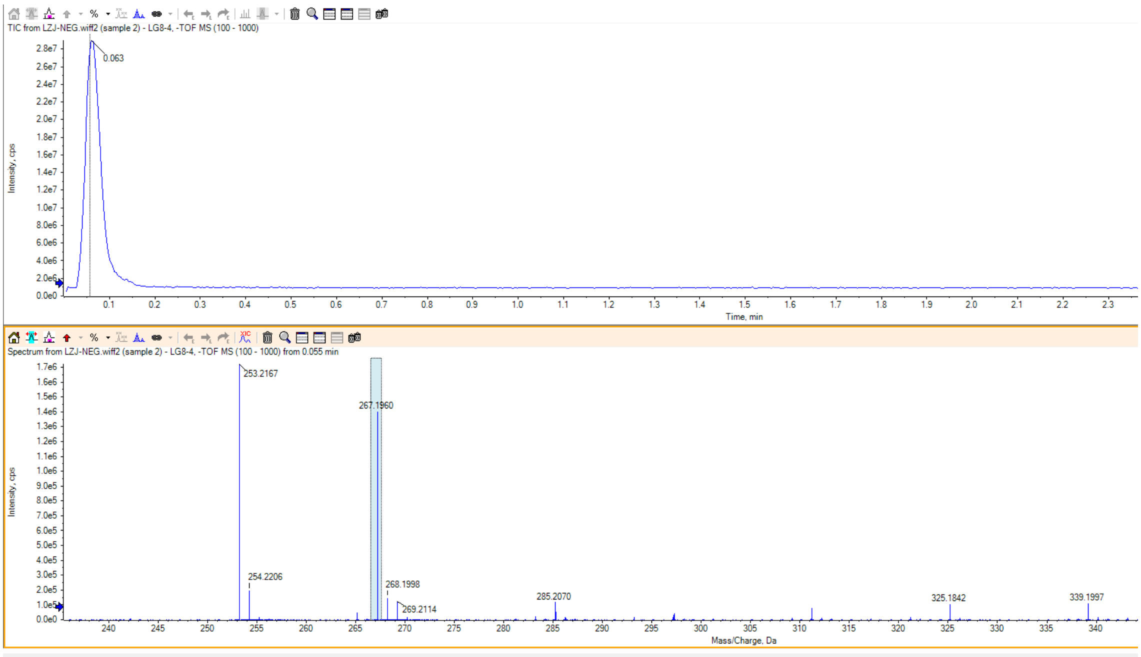

Figure S49. HRESI-MS spectrum of compound **15**

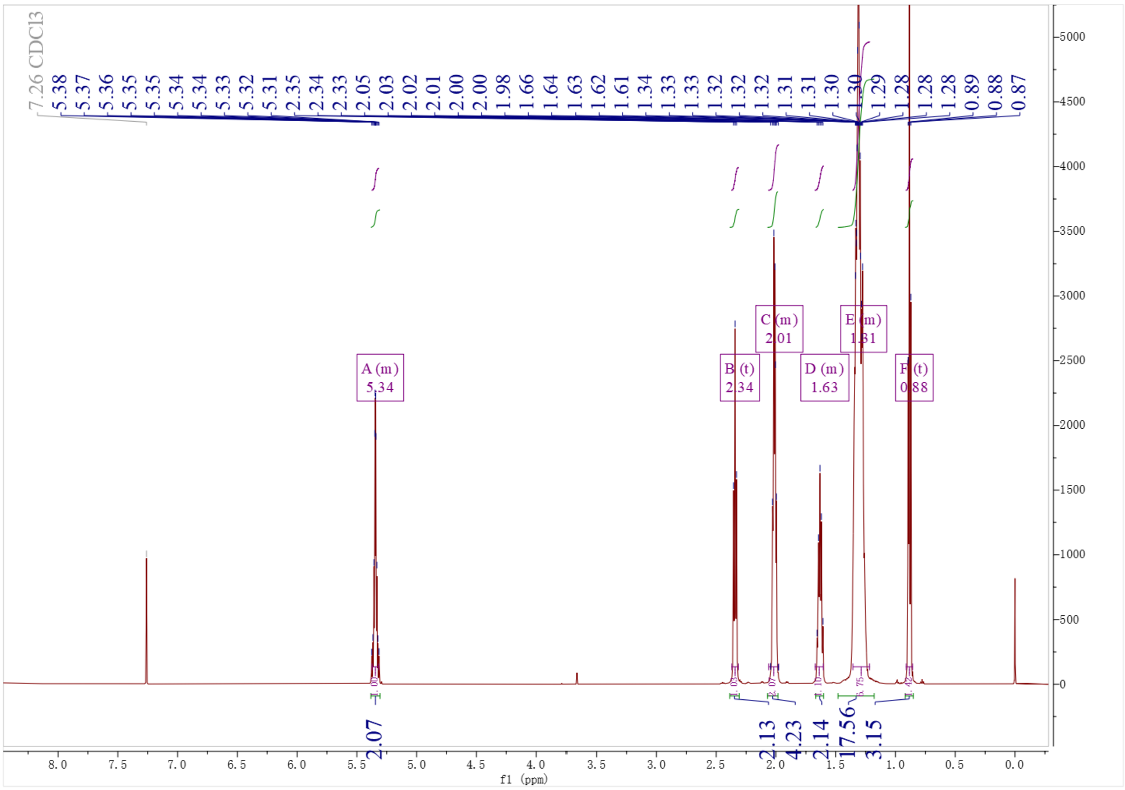

Figure S50. ^{1}H-NMR spectrum of compound **15** ($CDCl_3$, 600 MHz)

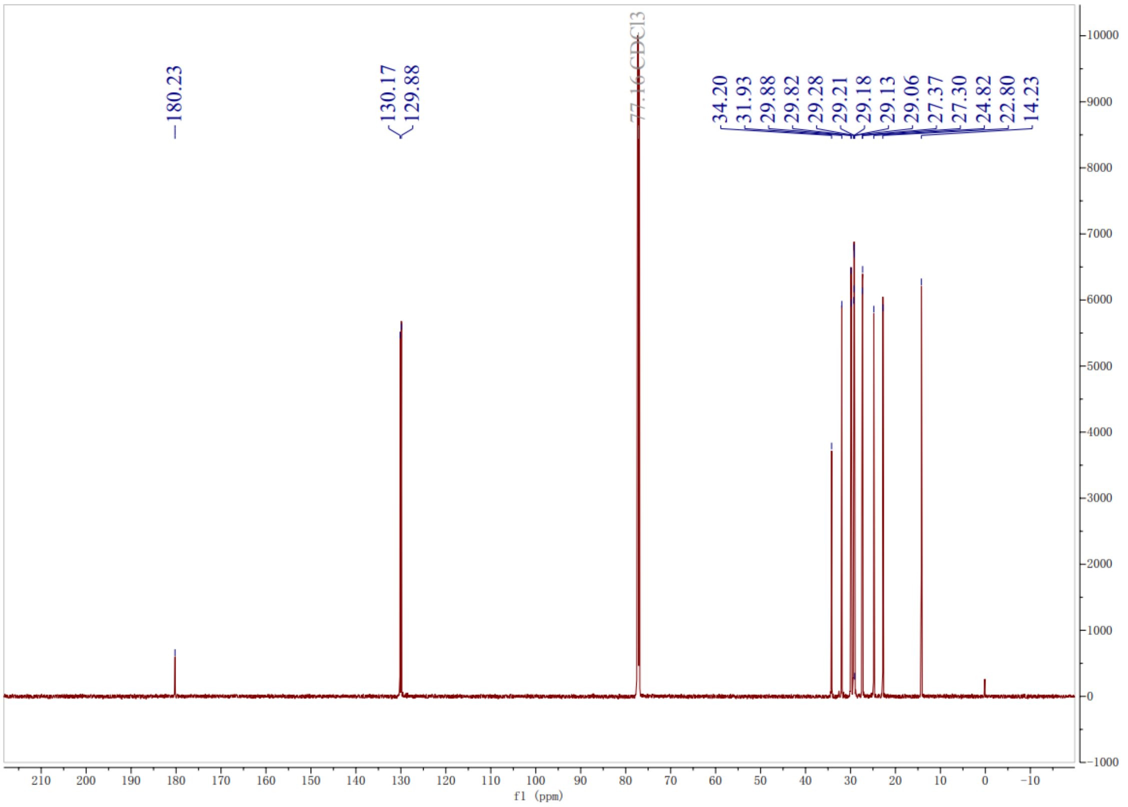

Figure S51. ^{13}C-NMR spectrum of compound **15** ($CDCl_3$, 150 MHz)

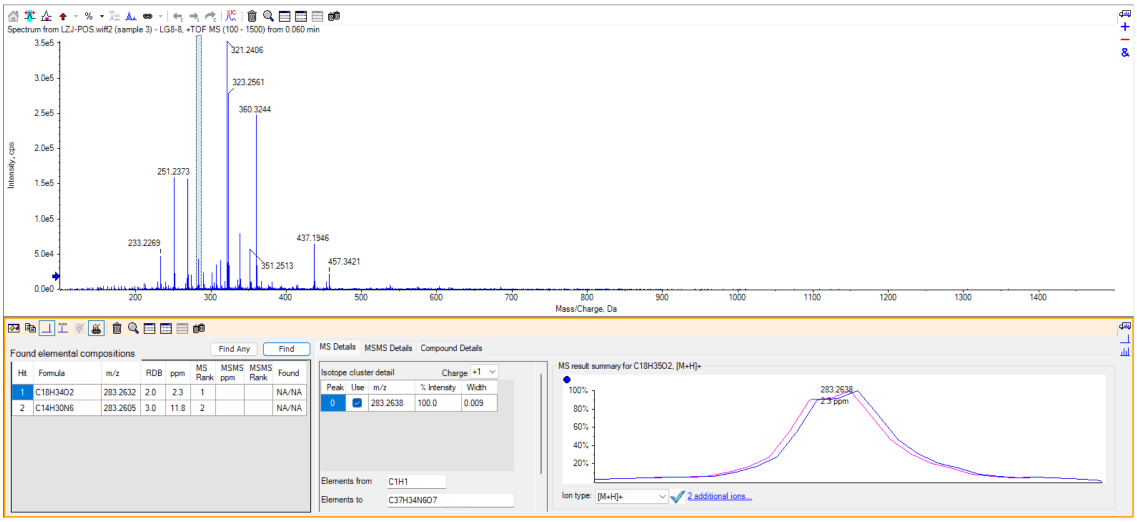

Figure S52. HRESI-MS spectrum of compound **16**

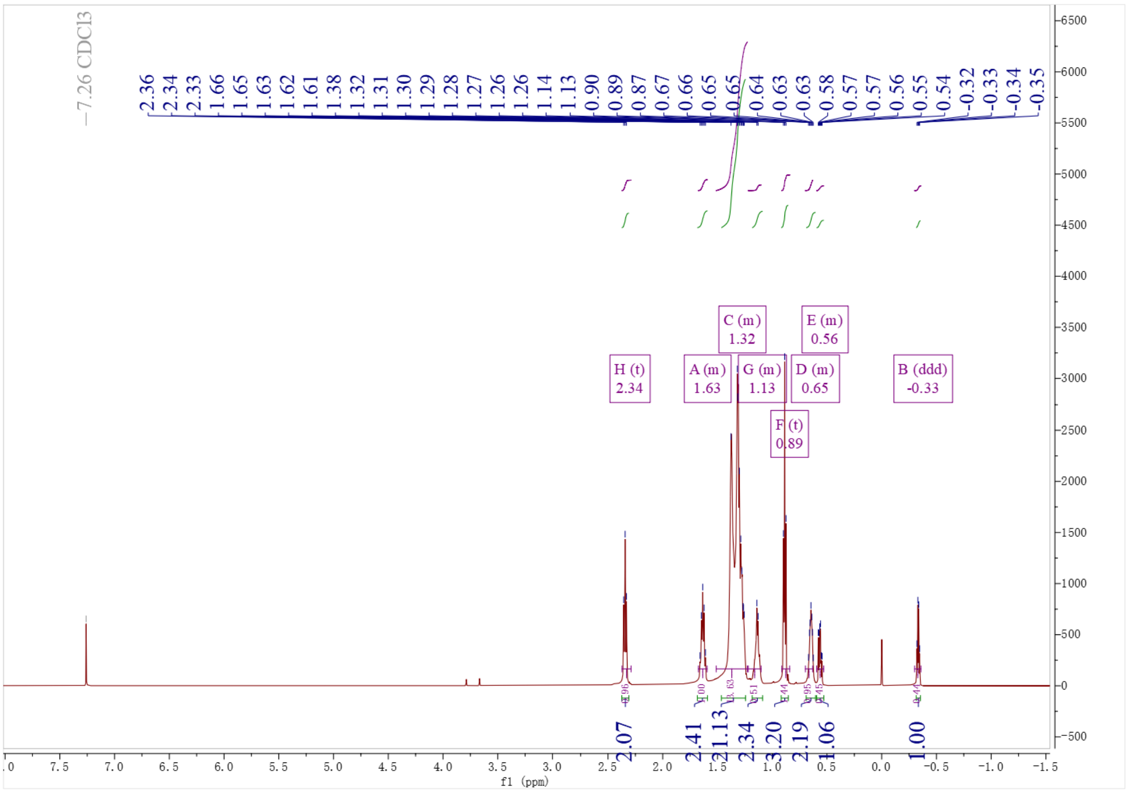

Figure S53. ^{1}H-NMR spectrum of compound **16** ($CDCl_3$, 600 MHz)

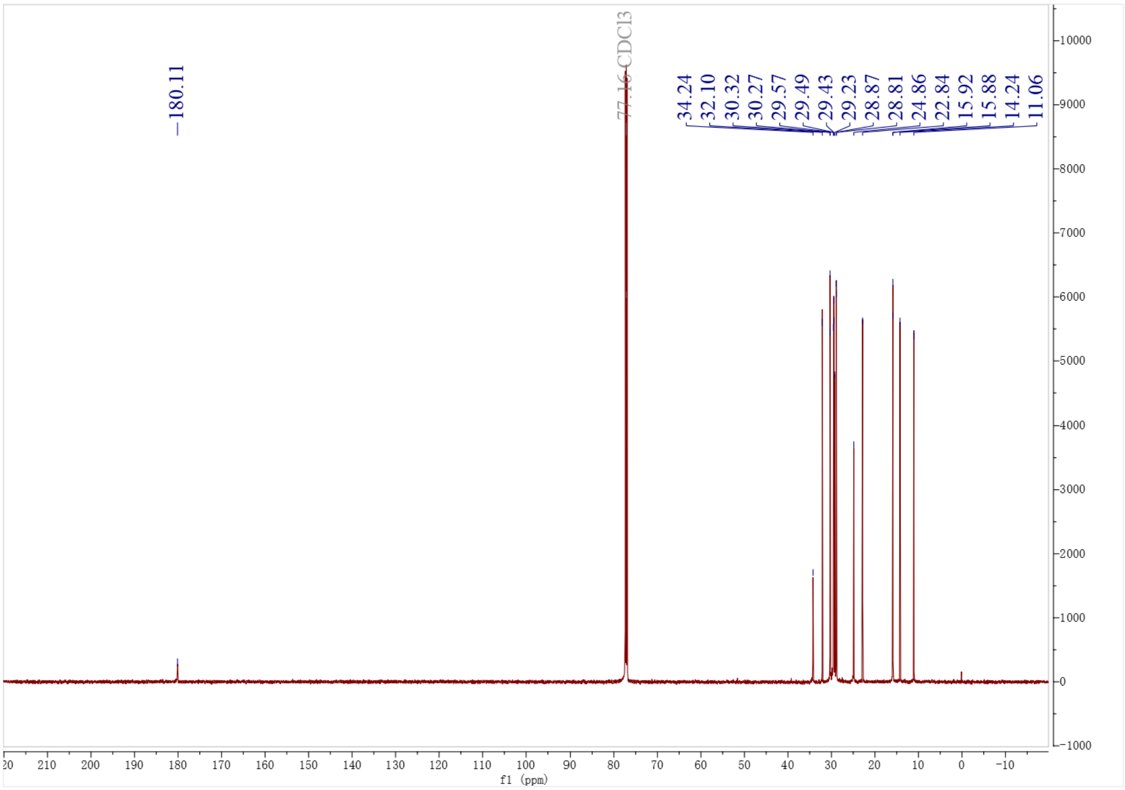

Figure S54. ^{13}C-NMR spectrum of compound **16** ($CDCl_3$, 150 MHz)

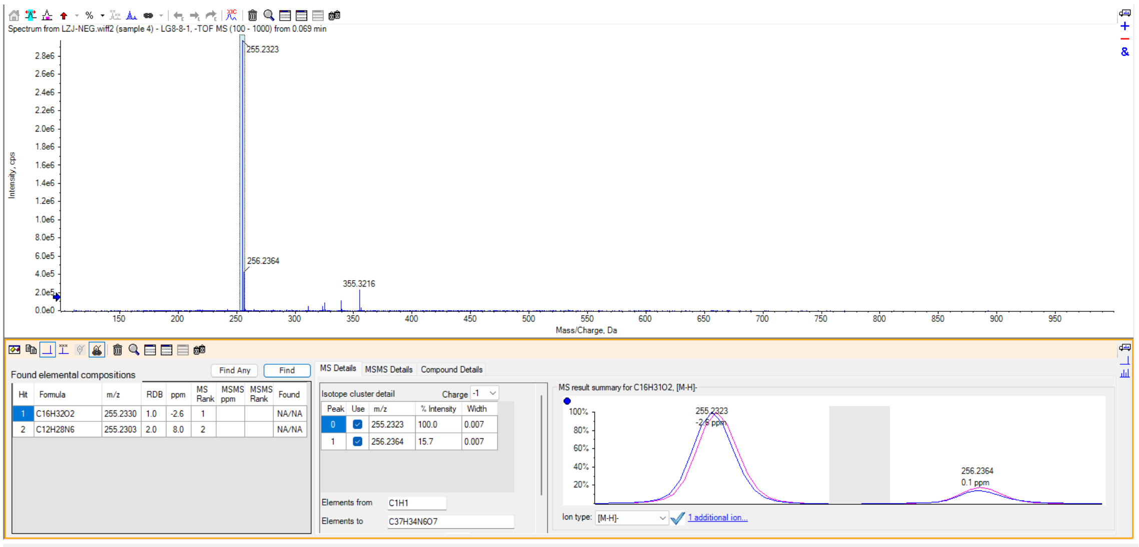

Figure S55. HRESI-MS spectrum of compound **17**

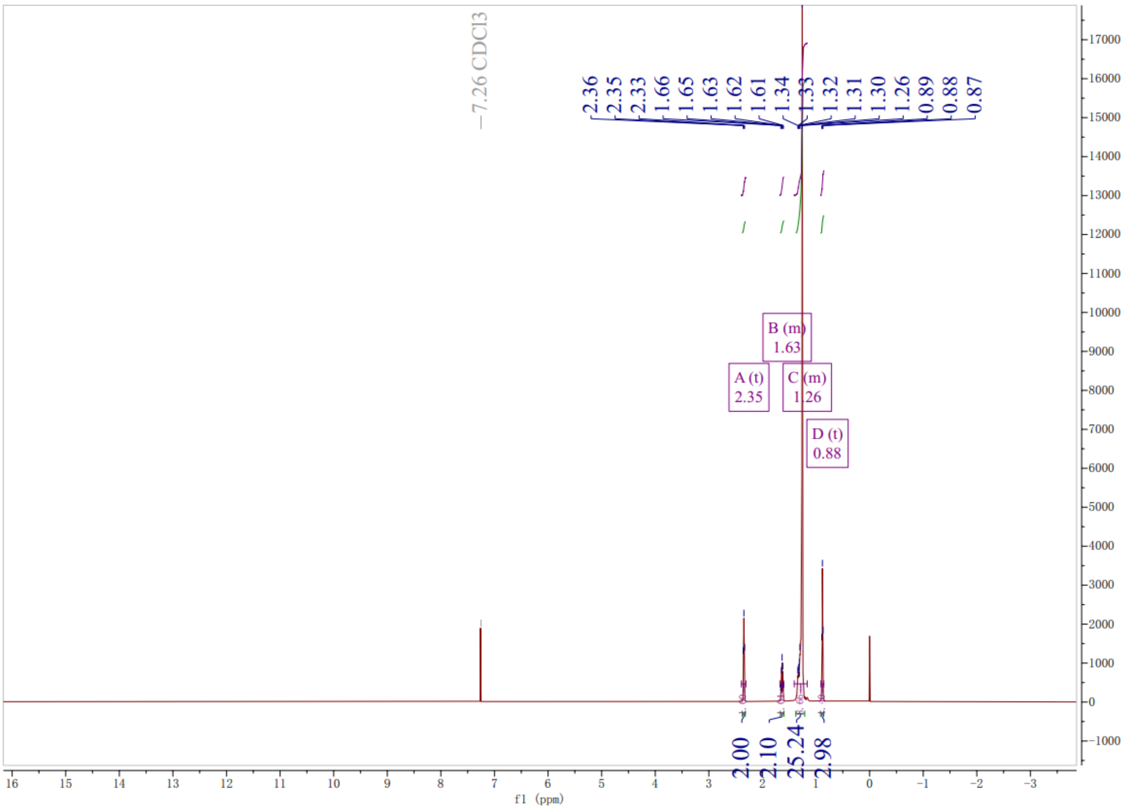

Figure S56. ^{1}H-NMR spectrum of compound **17** ($CDCl_3$, 600 MHz)

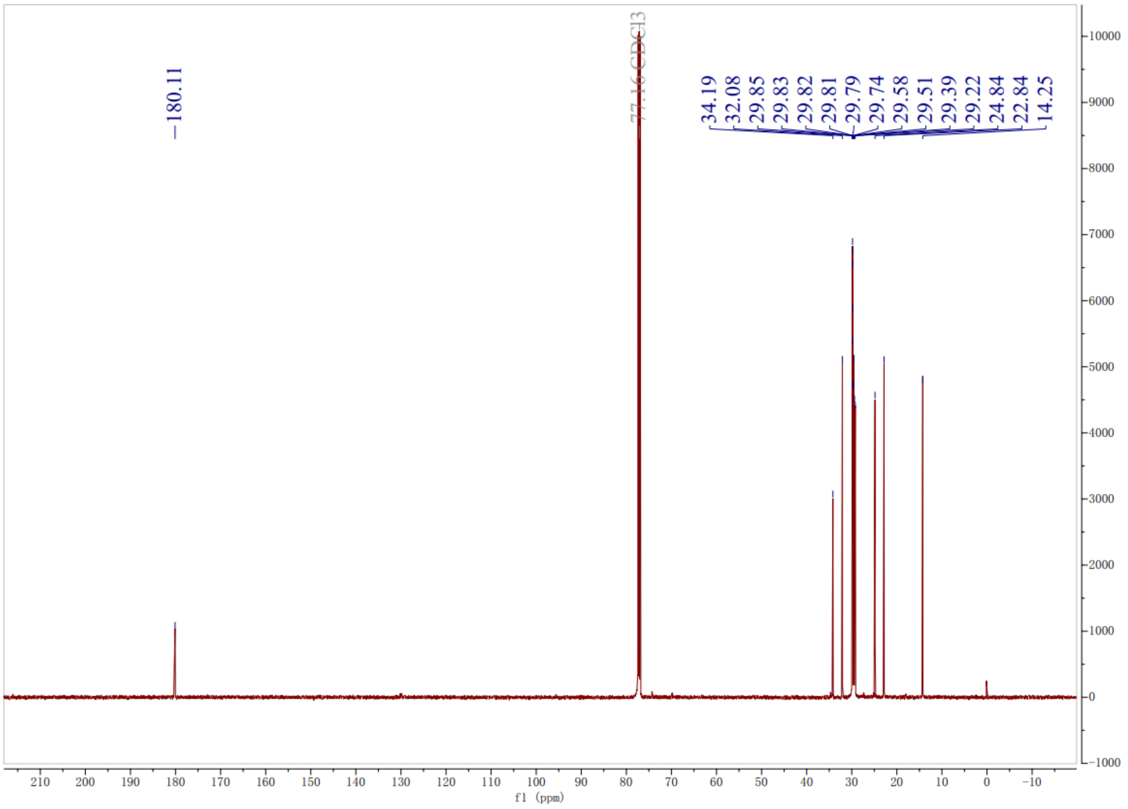

Figure S57. ^{13}C-NMR spectrum of compound **17** ($CDCl_3$, 150 MHz)

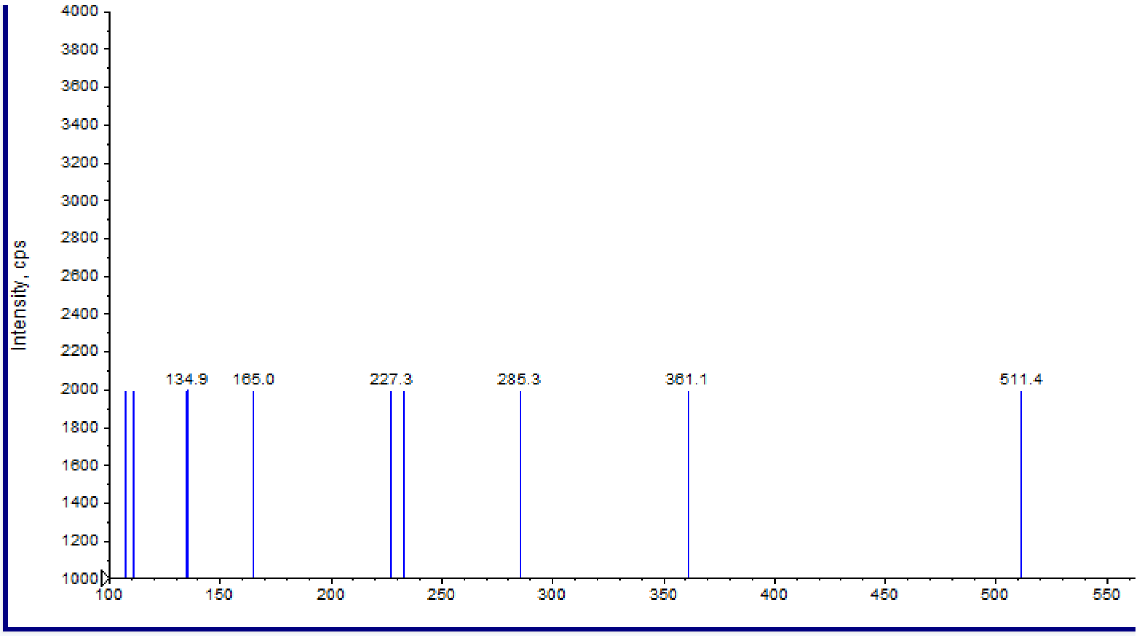

Figure S58. ESI-MS spectrum of compound **18**

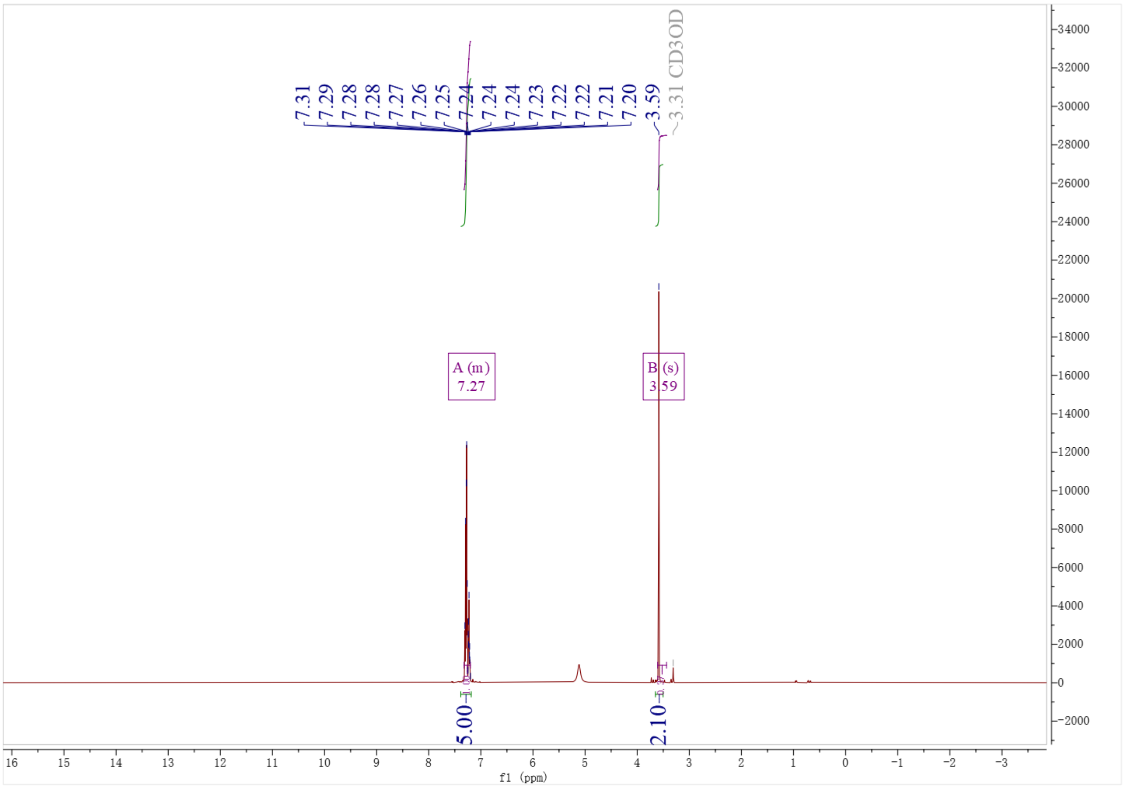

Figure S59. ^{1}H-NMR spectrum of compound **18** (CD_3OD, 600 MHz)

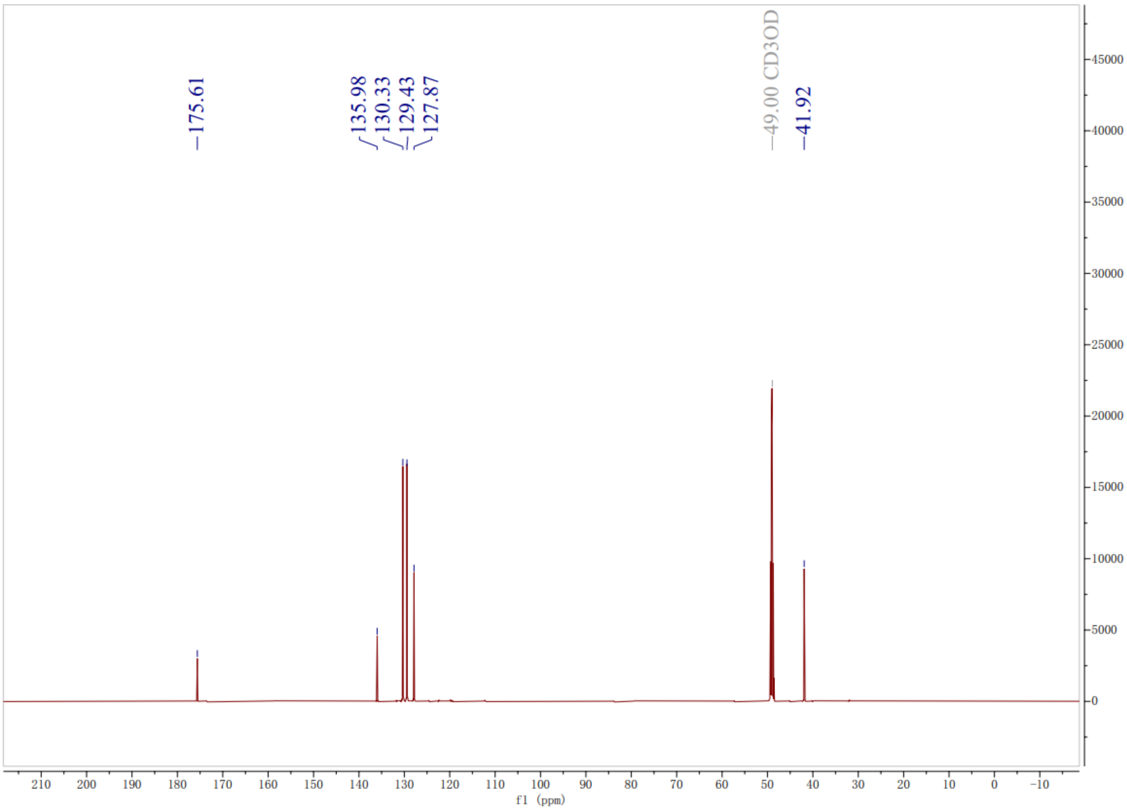

Figure S60. ^{13}C-NMR spectrum of compound **18** (CD_3OD, 150 MHz)

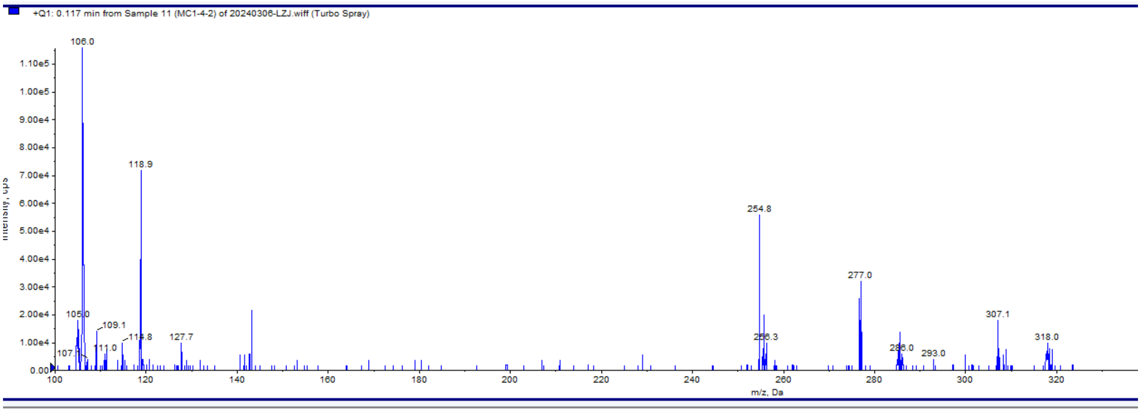

Figure S61. ESI-MS spectrum of compound **19**

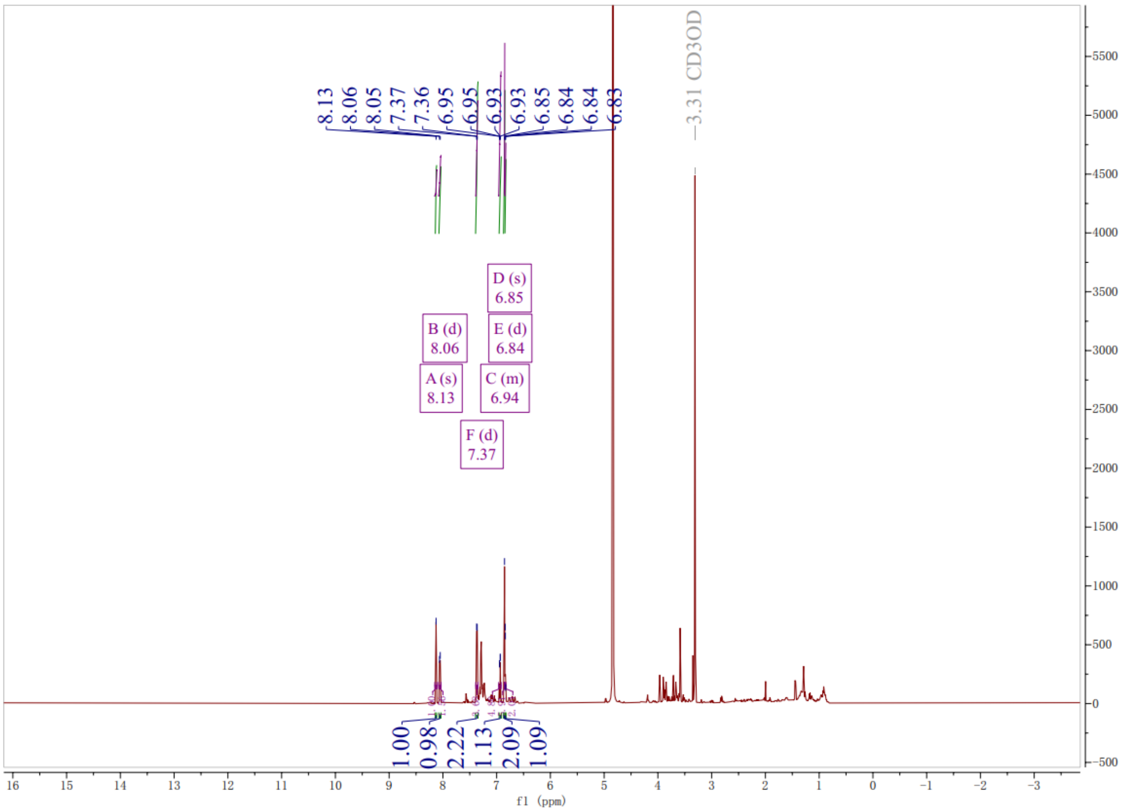

Figure S62. ^{1}H-NMR spectrum of compound **19** (CD_3OD, 600 MHz)

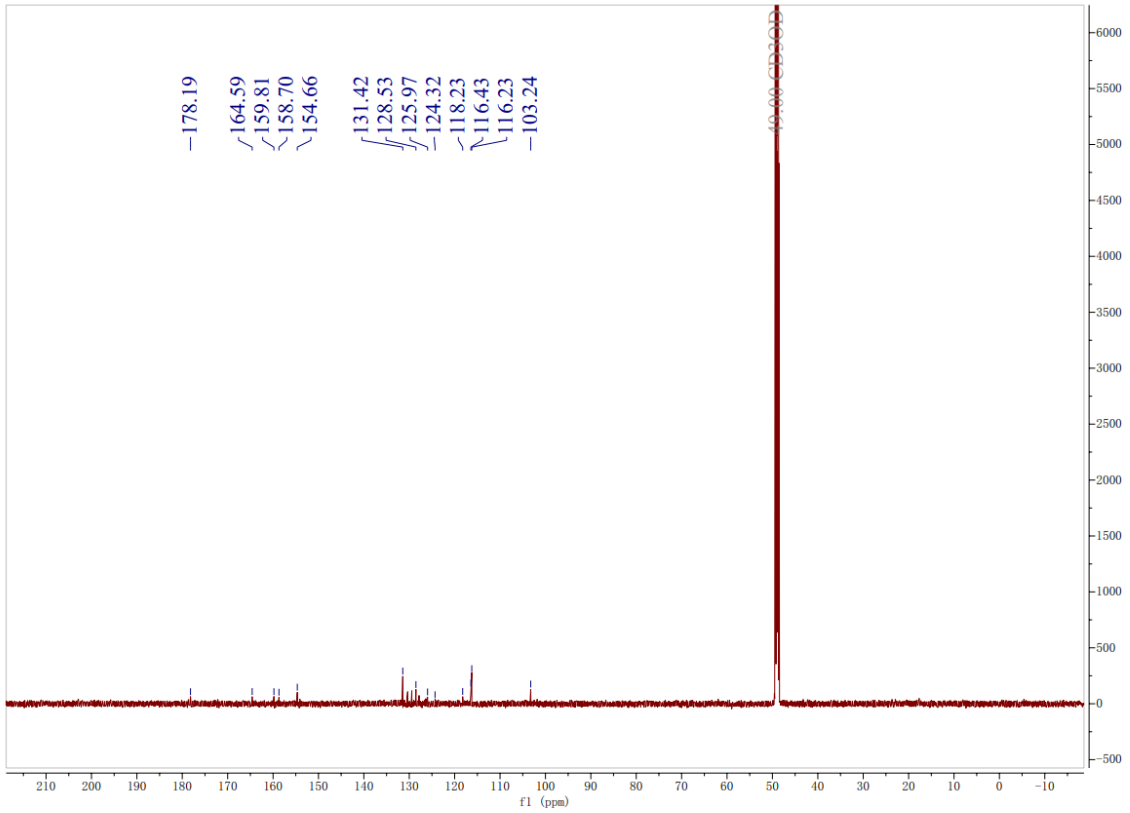

Figure S63. ^{13}C-NMR spectrum of compound **19** (CD_3OD, 150 MHz)

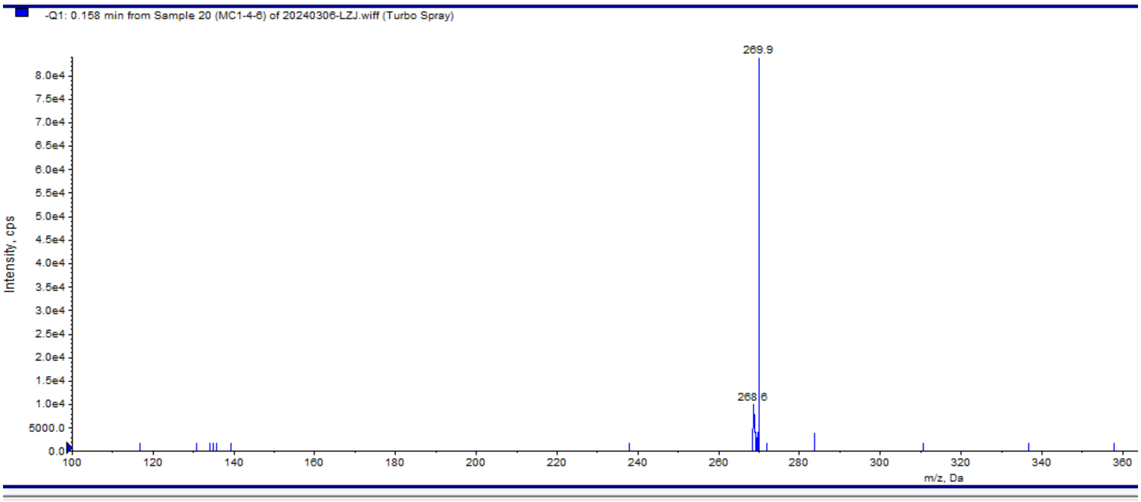

Figure S64. ESI-MS spectrum of compound **20**

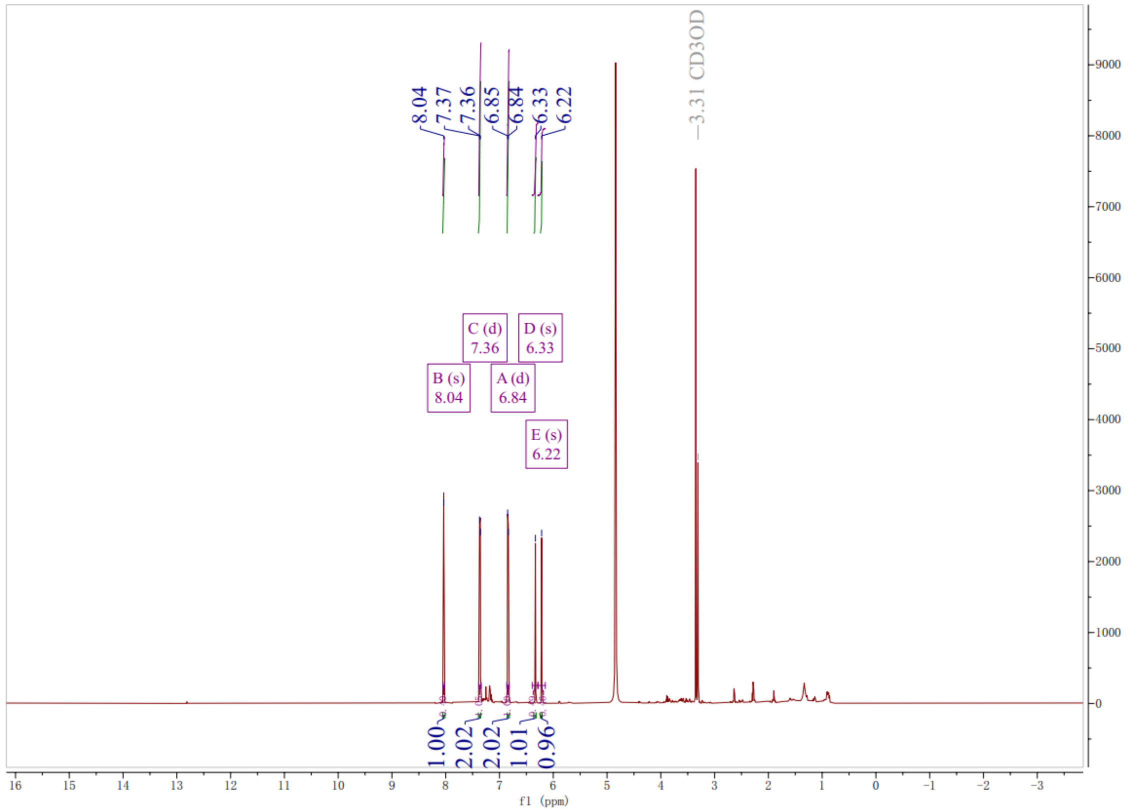

Figure S65. ^{1}H-NMR spectrum of compound **20** (CD_3OD, 600 MHz)

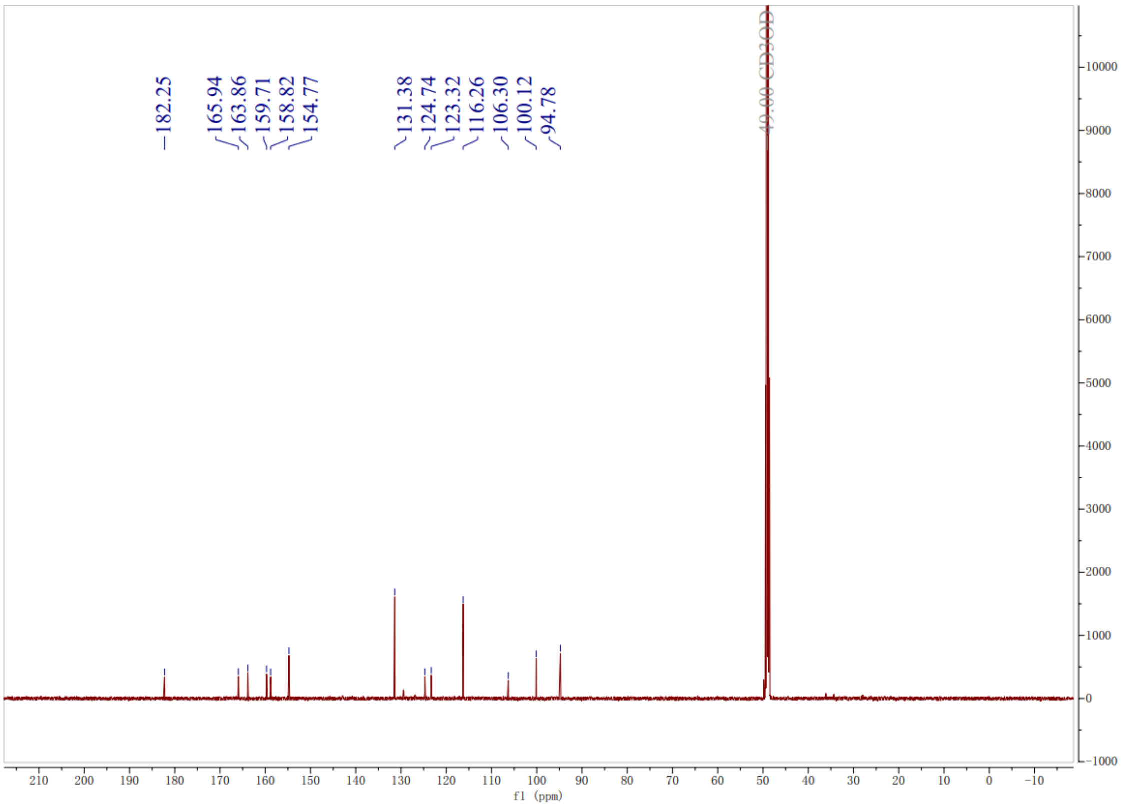

Figure S66. ^{13}C-NMR spectrum of compound **20** (CD_3OD, 150 MHz)

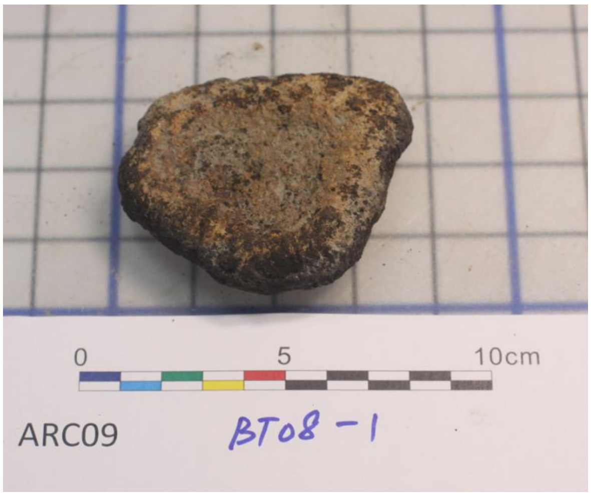

Figure S67. morphology of the ore sample from Arctic origin

References

1. Nakanishi, J.; Tatamidani, H.; Fukumoto, Y.; Chatani, N. A new synthesis of aldehydes by the palladium-catalyzed reaction of 2-pyridinyl esters with hydrosilanes. *ChemInform.* **2006**, *37*(31). https://doi.org/10.1002/chin.200631076
2. Mehnaz, S.; Saleem, R.S.; Yameen, B.; Pianet, I.; Schnakenburg, G.; Pietraszkiewicz, H.; Valeriote, F.; Josten, M.; Sahl, H.G.; Franzblau, S.G.; Gross, H. Lahorenoic acids A-C, ortho-dialkyl-substituted aromatic acids from the biocontrol strain Pseudomonas aurantiaca PB-St2. *J. Nat. Prod*. **2013**, *76*(2), 135-141. https://doi.org/10.1021/np3005166 PMID: 23402329.
3. Roemer, A.; Scholl, H.; Budzikiewicz, H.; Korth, H.; Pulverer, G. Bacterial constituents. part II. phenazines from pseudomonads. *Organische Chemie.* **1981**, *36B*(8), 1037-46.
4. Chen, C.; Pan, Y.; Zhao, H.; Xu, X.; Luo, Z.; Cao, L.; Xi, S.; Li, H.; Xu, L. Ruthenium(II)-catalyzed regioselective C-8 hydroxylation of 1,2,3,4-tetrahydroquinolines. *Org. Lett.* **2018**, *20*(21), 6799-6803. https://doi.org/10.1021/acs.orglett.8b02926 PMID: 30351962.
5. Li, C.; Wang, M.; Lu, X.; Zhang, L.; Jiang, J.; Zhang, L. Reusable brønsted acidic ionic liquid efficiently catalyzed N-formylation and *N*-acylation of amines. *ACS Sustain. Chem. Eng*. **2020**, *8*(11), 4353-4361.
6. Xu, H.J.; Liang, Y.F.; Cai, Z.Y.; Qi, H.X.; Yang, C.Y.; Feng, Y.S. CuI-nanoparticles-catalyzed selective synthesis of phenols, anilines, and thiophenols from aryl halides in aqueous solution. *J. Org. Chem*. **2001**, *76*(7), 2296-2300. https://doi.org/10.1021/jo102506x PMID: 21361386.
7. Hund, H.K., de Beyer, A.; Lingens, F. Microbial metabolism of quinoline and related compounds. VI. degradation of quinaldine by *Arthrobacter* sp. Biol. Chem. *Hoppe. Seyler.* **1990**, *371*(10), 1005-1008. https://doi.org/10.1515/bchm3.1990.371.2.1005 PMID: 2076195.
8. Suzuki, H.; Ohnishi, Y.; Furusho, Y.; Sakuda, S.; Horinouchi, S. Novel benzene ring biosynthesis from C(3) and C(4) primary metabolites by two enzymes. *J. Biol. Chem*. **2006**, *281*(48), 36944-36951. https://doi.org/10.1074/jbc.M608103200 PMID: 17003031.
9. Toyobo Co., Ltd. 3-Amino-4-hydroxybenzoic acid biosynthetic genes from *Streptomyces* griseus, and use in production of aminohydroxy aromatic carboxylic acid. JP2004283163, **2004**, 10-14.
10. Galm, U.; Dessoy, M. A.; Schmidt, J.; Wessjohann, L.A.; Heide, L. *In vitro* and *in vivo* production of new aminocoumarins by a combined biochemical, genetic, and synthetic approach. *Chem. Biol*. **2004**, *11*(2), 173-183. https://doi.org/10.1016/j.chembiol.2004.01.012 PMID: 15123279.
11. Higuchi, S.; Yasui, K. Interaction of N-6-substituted-9-methyladenines to poly-5-bromouridylic acid. *Nucleic Acids Symp. Ser.* **1983**, (12), 185-188.
12. Seim, K.L.; Obermeyer, A.C.; Francis, M.B. Oxidative modification of native protein residues using cerium(IV) ammonium nitrate. *J. Am. Chem. Soc.* **2001**, *133*(42), 16970-16976. https://doi.org/10.1021/ja206324q PMID: 21967510
13. Jiao, J.; Zhang, X.R.; Chang, N.H.; Wang, J.; Wei, J.F.; Shi, X.Y.; Chen, Z.G. A facile and practical copper powder-catalyzed, organic solvent-and ligand-free ullmann amination of aryl halides. *J. Org. Chem*. **2011**, *76*(4), 1180-1183. https://doi.org/10.1021/jo102169t PMID: 21261263
14. Senior, M.M.; Williamson, R.T; Martin, G.E. Using HMBC and adequate NMR data to define and differentiate long-range coupling pathways: Is the crews rule obsolete? *J. Nat. Prod.* **2013**, *76*(11), 2088-2093. https://doi.org/10.1021/np400562u PMID: 24195498
15. Choi, J.Y.; Choi E.H.; Jung H.W.; Oh, J.S.; Lee, W.H.; Lee, J. G.; Lee, S.H. Melanogenesis inhibitory compounds from saussureae radix. *Arch. Pharm. Res*. **2008**, *31*(3), 294-299. https://doi.org/10.1007/s12272-001-1154-0 PMID: 18409040
16. Mori, K.; Tashiro, T.; Akasaka, K.; Ohrui, H.; Fattorusso, E. Determination of the absolute configuration at the two cyclopropane moieties of plakoside A, an immunosuppressive marine galactosphingolipid. *Tetrahedron Letters* **2002**, *43*(20), 3719-3722. https://hub.uu2025.xyz/10.1016/S0040-4039(02)00606-8
17. Huang, X.A.; Yang, R.Z. A new hydroquinone diglucoside from Lysimachia fordiana. *Chem. Nat. Compd*. **2004**, *40*(5), 457-459. https://doi.org/10.1007/s10600-005-0010-5
18. Gutierrez-Lugo, M.T.; Woldemichael, G.M.; Singh, M.P.; Suarez, P.A.; Maiese, W.M.; Montenegro, G.; Timmermann, B.N. Isolation of three new naturally occurring compounds from the culture of *Micromonospora* sp. P1068. *Nat. Prod. Res*. **2005**, *19*(7),

645-652.
https://doi.org/10.1080/14786410412331272040 PMID: 16076633

19. He, J.; Fan, P.; Feng, S.; Shao, P.; Sun, P. Isolation and purification of two isoflavones from hericium erinaceum mycelium by high-speed counter-current chromatography. *Molecules* **2018**, *23*(3), 560. https://doi.org/10.3390/molecules23030560 PMID: 29498678
20. Selepe, M.A.; Drewes, S.E.; Van Heerden, F.R. Total synthesis of the pyranoisoflavone kraussianone 1 and related isoflavones. *J. Nat. Prod.* **2010**, *73*(10), 1680-1685.
